# Physiology and Pathophysiology in Ultra-Marathon Running

**DOI:** 10.3389/fphys.2018.00634

**Published:** 2018-06-01

**Authors:** Beat Knechtle, Pantelis T. Nikolaidis

**Affiliations:** ^1^Institute of Primary Care, University of Zurich, Zurich, Switzerland; ^2^Exercise Physiology Laboratory, Nikaia, Greece

**Keywords:** extreme endurance, pathophysiology, performance, injury, gender

## Abstract

In this overview, we summarize the findings of the literature with regards to physiology and pathophysiology of ultra-marathon running. The number of ultra-marathon races and the number of official finishers considerably increased in the last decades especially due to the increased number of female and age-group runners. A typical ultra-marathoner is male, married, well-educated, and ~45 years old. Female ultra-marathoners account for ~20% of the total number of finishers. Ultra-marathoners are older and have a larger weekly training volume, but run more slowly during training compared to marathoners. Previous experience (e.g., number of finishes in ultra-marathon races and personal best marathon time) is the most important predictor variable for a successful ultra-marathon performance followed by specific anthropometric (e.g., low body mass index, BMI, and low body fat) and training (e.g., high volume and running speed during training) characteristics. Women are slower than men, but the sex difference in performance decreased in recent years to ~10–20% depending upon the length of the ultra-marathon. The fastest ultra-marathon race times are generally achieved at the age of 35–45 years or older for both women and men, and the age of peak performance increases with increasing race distance or duration. An ultra-marathon leads to an energy deficit resulting in a reduction of both body fat and skeletal muscle mass. An ultra-marathon in combination with other risk factors, such as extreme weather conditions (either heat or cold) or the country where the race is held, can lead to exercise-associated hyponatremia. An ultra-marathon can also lead to changes in biomarkers indicating a pathological process in specific organs or organ systems such as skeletal muscles, heart, liver, kidney, immune and endocrine system. These changes are usually temporary, depending on intensity and duration of the performance, and usually normalize after the race. In longer ultra-marathons, ~50–60% of the participants experience musculoskeletal problems. The most common injuries in ultra-marathoners involve the lower limb, such as the ankle and the knee. An ultra-marathon can lead to an increase in creatine-kinase to values of 100,000–200,000 U/l depending upon the fitness level of the athlete and the length of the race. Furthermore, an ultra-marathon can lead to changes in the heart as shown by changes in cardiac biomarkers, electro- and echocardiography. Ultra-marathoners often suffer from digestive problems and gastrointestinal bleeding after an ultra-marathon is not uncommon. Liver enzymes can also considerably increase during an ultra-marathon. An ultra-marathon often leads to a temporary reduction in renal function. Ultra-marathoners often suffer from upper respiratory infections after an ultra-marathon. Considering the increased number of participants in ultra-marathons, the findings of the present review would have practical applications for a large number of sports scientists and sports medicine practitioners working in this field.

## Introduction

In recent years and decades, races longer than the classical marathon distance (42.195 km) have experienced a boom. With the increase in the number of races and finishers (Nikolaidis and Knechtle, 2018a), ultra-marathons—defined as any running distance longer than the marathon distance—became more and more interesting for scientific research.

The aim of this narrative review is to present the current state of knowledge on the topic of physiology and pathophysiology of ultra-marathon running. For this purpose, we have searched all scientific papers on ultra-marathon in Scopus (www.scopus.com) until March 2018. The keywords were “ultra-marathon” and “ultra marathon.” This search resulted in more than 700 articles. All articles were then sorted so that only studies (i.e., case reports and original papers) on running were taken into consideration. Studies investigating swimmers, cyclists, triathletes or other sports were excluded. Specifically, studies with a practical relevance for the athlete, the coach and the physician were considered.

## What is an ultramarathon?

**Figure 1:**
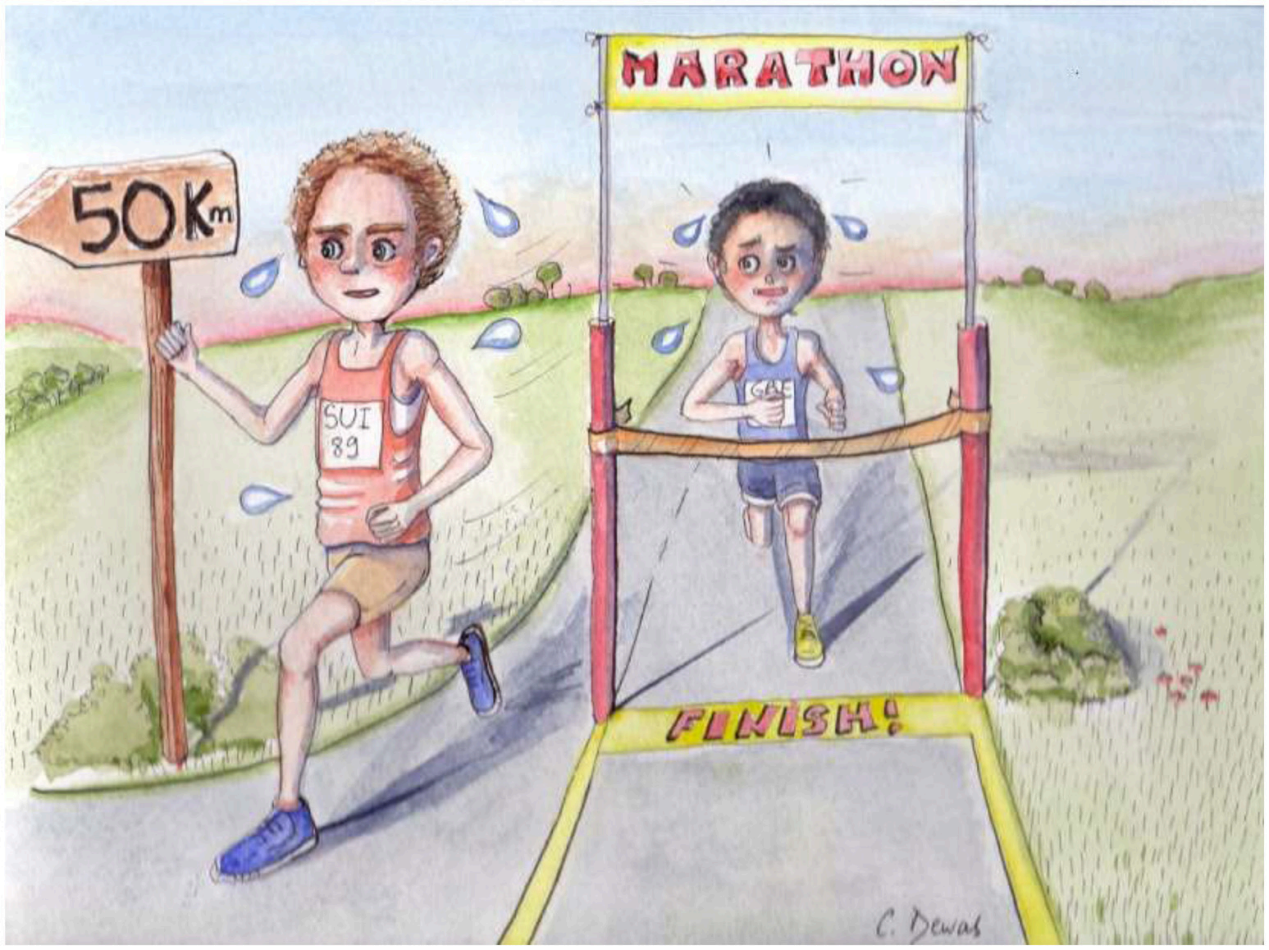
A 50-km ultra-marathon is the shortest ultra-marathon. Figure by Céline Dewas.

An ultra-marathon is any running event where the running distance is longer than the traditional race length of a marathon of 42.195 km. The shortest ultra-marathon is the 50-km run (Figure 1). An ultra-marathon can also be defined as a running competition lasting 6 h in duration or longer (Zaryski and Smith, 2005). Ultra-marathons are performed as distance-limited runs held in km or miles or as time-limited events held in hours or days (www.ultramarathonrunning.com). The most frequently performed ultra-marathons held as a distance-limited events are 50-km, 100-km, 50-miles, and 100-miles ultra-marathons (www.ultra-marathon.org). In addition, there are a variety of races of different distances, with the longest ultra-marathons up to 1,000 km for races held in km and 3,100 miles for races held in miles (www.ultra-marathon.org). In time-limited ultra-marathons, runners are competing mainly during 6, 12, 24, 48, 72 h, 6 and 10 days (www.ultra-marathon.org). Ultra-marathon running also includes multi-stage races, such as crossing countries or even continents (www.ultra-marathon.org). The longest official ultra-marathon in the world, which takes place regularly, is the “Self-Transcendence 3,100 Mile Race” covering the total distance of 3,100 miles (4,989 km) (www.3100.ws).

An analysis of all 100-miles runners from 1977 to 2008 showed that both the number of races offered and the number of official finishers increased exponentially (Hoffman et al., 2010a). Surprisingly, running performance has not improved over all these years (Hoffman, 2010), which should be attributed to the increased number of finishers. That is, more “recreational” runners participate nowadays compared to fewer and more “competitive” runners in the past.

## Who are ultra-marathoners?

With regards to their socio-demographic characteristics of US ultra-marathoners in particular (Tokudome et al., 2004; Hoffman and Krishnan, 2013), 100-mile ultra-marathoners were ~44.5 years old, usually men (80.2%), mostly married (70.1%) (Hoffman et al., 2014) and had a bachelor (43.6%) or higher (37.2%) university degree (Hoffman and Fogard, 2012). Ultra-marathoners have a stable body weight during their lifetime and gain less body weight with increasing age (Hoffman and Fogard, 2012; Hoffman et al., 2014) compared to the general population. The reason is likely to be the low BMI at a younger age (Hoffman et al., 2014).

The average age at the first participation in an ultra-marathon was ~36 years and before the first start in an ultra-marathoners had an experience of ~7 years of competition in shorter distances (Hoffman and Krishnan, 2013). The average age did not change at the first start in an ultra-marathon in recent decades, the ultra-marathoners completed more than 3,000 km in the year before an important competition and the training kilometers correlated with the length of the longest ultra-marathon that they completed (Hoffman and Krishnan, 2013). Ultra-marathoners were healthier compared to the general population and less often absent at school or at work. Serious diseases such as cancer (~4.5%), coronary heart disease (~0.7%), strokes (~0.7%), diabetes (~0.7%), and HIV (~0.2%) were very rare in ultra-marathoners, who, on the other hand, had more allergies (~25.1%) and exercise-induced asthma (~13.0%) than the general population (Hoffman and Krishnan, 2014).

In general, ultra-marathoners are highly intrinsically motivated people (Kruger and Saayman, 2013) and have a very healthy lifestyle (Tokudome et al., 2004). Ultra-marathoners do not seem to be motivated by the competition as such, but rather by the adventure ultra-marathon (Doppelmayr and Molkenthin, 2004). For an ultra-marathoner, the following five aspects are central for a successful race: the preparation and the strategy, the management of the race, the discovery of the performance, the personal performance and the shared experience with the opponents (Simpson et al., 2014).

Although it is well-known that ultra-marathon running can lead to adverse aspects regarding health, a large part of ultra-marathoners would not stop if they learned it was bad for their health. These ultra-marathoners were younger and less likely to be married runners with less children and a lower health orientation but with high personal goal achievement, psychological coping, and life meaning scores (Hoffman and Krouse, 2018).

When we consider the the winner list of the “Self-Transcendence 3,100 Mile Race” (Table 1) and world records in ultra-marathon running of elite and age group runners (Tables 2–6), we inevitably notice that there are “endurance talents” or “exceptional runners” in ultra-marathon running. It is striking that the Greek Yiannis Kouros has a large number of world records over different distances and in different age groups. His list of victories in ultra-marathons and world records is almost endless (www.yianniskouros.gr/index.php/en/kourosvictories). He is considered as one of the best runners in the world and the best ultra-marathoner of all time.

**Table 1:** Winners in the “Self-Transcendence 3100 Mile Race” in New York, USA, since1997, from https://3100.srichinmoyraces.org/.

**Edition**	**Men**	**Race time (d:h:min:s)**	**Women**	**Race time (d:h:min:s)**
1997	Edward Kelley (USA)	47:15:19:56	Suprabha Beckjord (USA)	51:02:09:56
1998	István Sipos (Hungary)	46:17:02:06	Suprabha Beckjord (USA)	49:14:30:54
1999	Edward Kelley (USA)	48:12:42:46	Suprabha Beckjord (USA)	51:14:16:17
2000	Asprihanal Aalto (Finland)	47:13:29:55	Suprabha Beckjord (USA)	54:15:51:34
2001	Asprihanal Aalto (Finland)	48:10:56:12	Suprabha Beckjord (USA)	52:10:37:42
2002	Madhupran Wolfgang Schwerk (Germany)	42:13:24:03	Suprabha Beckjord (USA)	51:12:08:06
2003	Namitabha Arsic (Serbia)	49:02:24:45	Suprabha Beckjord (USA)	56:03:00:22
2004	Asprihanal Aalto (Finland)	46:06:55:11	Suprabha Beckjord (USA)	55:13:13:00
2005	Srdjan Stojanovich (Serbia)	46:10:51:16	Suprabha Beckjord (USA)	63:04:23:28
2006	Madhupran Wolfgang Schwerk (Germany)	41:08:16:29	Suprabha Beckjord (USA)	60:04:35:24
2007	Asprihanal Aalto (Finland)	43:04:26:32	Suprabha Beckjord (USA)	58:07:54:27
2008	Asprihanal Aalto (Finland)	44:02:42:15	Suprabha Beckjord (USA)	56:17:51:22
2009	Asprihanal Aalto (Finland)	43:16:28:06	Suprabha Beckjord (USA)	60:08:58:51
2010	Asprihanal Aalto (Finland)	46:07:37:24	—	—
2011	Sarvagata Ukrainskyi (Ukraine)	44:13:38:52	Surasa Mairer (Austria)	53:15:54:25
2012	Grahak Cunningham (Australia)	43:10:36:39	—	—
2013	Vasu Duzhiy (Russia)	47:05:39:00	Surasa Mairer (Austria)	50:04:57:24
2014	Sarvagata Ukrainskyi (Ukraine)	44:06:58:10	Sarah Barnett (Australia)	50:03:55:08
2015	Asprihanal Aalto (Finland)	40:09:06:21	Surasa Mairer (Austria)	49:07:52:24
2016	Yuri Trostenyuk (Ukraine)	46:01:10:25	Kaneenika Janakova (Slovakia)	51:07:31:07
2017	Vasu Duzhiy (Russia)	46:17:38:22	Kaneenika Janakova (Slovakia)	48:14:24:10

**Table 2:** World records in female elite and age group athletes in distance-limited races, from http://www.iau-ultramarathon.org/.

	**h:min:s**	**Name**	**Origin**	**Year**
**50 km**				
World record	03:08:39	Van der Merwe Frith	South Africa	1989
35–39	03:15:43	Harrison Susan	Great Britain	2010
40–44	03:17:30	Gooderham Emma	Great Britain	2011
45–49	03:32:24	Kiddy Sandra	USA	1983
50–54	03:41:57	Petrie Lavina	Great Britain	1994
55–59	03:56:55	Kiddy Sandra	USA	1992
60–64	04:13:32	Torpy Tina	Australia	2007
65–69	04:38:22	Young Shirley	Australia	1995
70–74	04:48:23	Rhodes Myra	USA	2003
75–79	06:05:16	Klein Helen	USA	2002
80–84	05:57:53	Klein Helen	USA	2003
**100 km**				
World record	06:33:11	Abe Tomoe	Japan	2000
35–39	07:00:27	Sakurai Norimi	Japan	2007
40–44	07:20:22	Auxiliadora Maria	Brazil	1998
45–49	07:43:55	Nagy Judit	Hungary	2012
50–54	07:51:10	Arbogast	USA	2011
55–59	08:39:52	Braun Marion	Germany	2012
60–64	09:20:07	Schmitz Ursula	Germany	1994
65–69	10:21:21	Schmitz Ursula	Germany	1999
70–74	11:36:17	Young Shirley	Australia	2002
75–79	17:51:57	Dinges, Ursula	Germany	2017
80–84	17:19:18	Noyel Marie-Claude	France	2000
85–89	18:15:17	Noyel Marie-Claude	France	2002
**100 miles**				
World record	13:45:49	Slaby Gina	USA	2016
35–39	13:45:49	Slaby Gina	USA	2016
40–44	14:09:43	Smith Pam	USA	2016
45–49	13:52:07	Kudo Mami	Japan	2011
50–54	15:02:30	Lomsky	Germany	1993
55–59	17:13:42	Trapp Sue	USA	2002
60–64	20:47:35	Lamothe Francoise	France	1986
65–69	21:03:01	Klein Helen	USA	1992
70–74	23:29:34	Klein Helen	USA	1993
75–79	45:55:46	Biondic, Erlinda	Canada	2017
80–84	47:21:12	Macklow Barbara	USA	2016
	**d:h:min:s**	**Name**	**Origin**	**Year**
**1,000 km**				
World record	7:01:28:29	Robinson Eleanor	Great Britain	1998
35–39	8:10:03:37	BarwickSandra	New Zealand	1988
40–44	7:16:08:37	Mairer Paula	Austria	2002
45–49	8:12:06:20	Brown Sandra	Great Britain	1996
50–54	8:00:27:06	Robinson Eleanor	Great Britain	1998
55–59	9:17:15:53	Vollmerhausen Christel	Germany	1990
**1,000 miles**				
World record	12:14:38:40	BarwickSandra	New Zealand	1991
35–39	14:06:15:25	Locs Antana	Canada	1994
40–44	12:14:38:40	BarwickSandra	New Zealand	1991
45–49	14:10:27:21	Brown Sandra	Great Britain	1996
50–54	13:01:54:02	Robinson Eleanor	Great Britain	1998
55–59	16:01:59:40	Vollmerhausen Christel	Germany	1990
60–64	17:19:38:00	Khisamutdinova Svetlana	Russia	2004

**Table 3:** World records in male elite and age group athletes in distance-limited races, from http://www.iau-ultramarathon.org/.

	**h:min:s**	**Name**	**Origin**	**Year**
**50 km**				
World record	02:43:38	Magawana Thompson	South Africa	1988
35–39	02:43:38	Magawana Thompson	South Africa	1988
40–44	02:48:39	Thys Gert	South Africa	2012
45–49	03:04:48	Moore Stephen	Great Britain	1994
50–54	02:58:18	Kotov Vladimir	Belarus	2010
55–59	03:17:26	Perkins Otho	USA	1986
60–64	03:29:51	Remmele Erwin	Germany	1999
65–69	03:41:41	Gillis Malcolm	USA	1998
70–74	04:15:55	Hofmann Wilhelm	Germany	1997
75–79	04:14:57	Gutbier Heinrich	Germany	1999
80–84	05:36:41	Simon Josef	Luxemburg	2015
**100 km**				
World record	06:10:20	Ritchie Donald	Great Britain	1978
35–39	06:10:20	Ritchie Donald	Great Britain	1978
40–44	06:18:24	Ardemagni Mario	Italy	2004
45–49	06:30:35	Vuillemenot Roland	France	1995
50–54	06:43:33	Vuillemenot Roland	France	1996
55–59	07:10:51	Engeländer Karl	Germany	1992
60–64	07:53:43	Juckel Bernd	Germany	2012
65–69	08:07:22	Van der Lee Will	Netherlands	1995
70–74	08:53:45	Courtillon Max	France	1997
75–79	09:43:51	Gutbier Heinrich	Germany	1999
80–84	10:40:43	Caponetto Antonino	Italy	2012
85–89	21:06:25	Schauer Johann	Switzerland	1996
90–94	18:09:04	Fagnani Walter	Italy	2015
**100 miles**				
World record	11:28:03	Kharitonov Oleg	Russia	2002
35–39	11:28:03	Kharitonov Oleg	Russia	2002
40–44	12:00:00	Kouros Yiannis	Greece	1997
45–49	12:19:11	Kouros Yiannis	Greece	2001
50–54	13:52:29	Aldous Jay	USA	2011
55–59	15:14:35	Cooper Dave	Great Britain	1990
60–64	14:37:54	Young Cliff	Australia	1983
65–69	17:08:15	Mainix Christian	France	2004
70–74	18:16:49	Jones Max	Great Britain	1997
75–79	20:43:49	Oliver Geoffrey	Great Britain	2009
80–84	64:46:08	Baglione Daniel	USA	2014
**100 km**				
World record	06:10:20	Ritchie Donald	Great Britain	1978
35–39	06:10:20	Ritchie Donald	Great Britain	1978
40–44	06:18:24	Ardemagni Mario	Italy	2004
45–49	06:30:35	Vuillemenot Roland	France	1995
50–54	06:43:33	Vuillemenot Roland	France	1996
55–59	07:10:51	Engeländer Karl	Germany	1992
60–64	07:53:43	Juckel Bernd	Germany	2012
65–69	08:07:22	Van der Lee Will	Netherlands	1995
70–74	08:53:45	Courtillon Max	France	1997
75–79	09:43:51	Gutbier Heinrich	Germany	1999
80–84	10:40:43	Caponetto Antonino	Italy	2012
85–89	21:06:25	Schauer Johann	Switzerland	1996
90–94	18:09:04	Fagnani Walter	Italy	2015
	**d:h:min:s**	**Name**	**Origin**	**Year**
**1,000 km**				
World record	5:16:17:00	Kouros Yiannis	Greece	1984
40–49	7:11:40:15	Brown Richard	USA	1988
45–49	5:17:38:52	Kouros Yiannis	Greece	2005
50–54	6:23:32:09	Schwerk Wolfgang	Germany	2010
55–59	5:22:52:58	Mainix Gilbert	France	1992
**1,000 miles**				
World record	10:10:30:36	Kouros Yiannis	Greece	1988
35–39	10:10:30:36	Kouros Yiannis	Greece	1988
40–44	12:22:35:53	Bauer Siggy	New Zealand	1988
45–49	12:01:42:52	Howie Al	Great Britain	1991
50–54	11:23:18:10	Schwerk Wolfgang	Germany	2010
55–59	11:13:54:58	Silkinas Petras	Lithuania	1998

**Table 4:** World records in women in elite and age group athletes in time-limited races, from http://www.iau-ultramarathon.org/.

	**km**	**Name**	**Origin**	**Year**
**6-h-run**				
World record	83.275	Alder-Baerens Nele	Germany	2016
35–39	83.275	Alder-Baerens Nele	Germany	2016
40–44	79.000	Auxiliadora Maria	Brazil	2000
45–49	77.600	Jouault Huguette	France	1996
50–54	72,341	Stöppler Simone	Germany	2016
55–59	71.371	Agesta Eva	Spain	2015
60–64	65.244	Eremina Galina	Russia	2013
65–69	62.658	Young Shirley	Australia	1999
70–74	60.992	Young Shirley	Australia	2002
75–79	39.329	Dinges, Ursula	Germany	2004
**12-h-run**				
World record	149.130	Herron Camille	USA	2017
35–39	149.130	Herron Camille	USA	2017
40–44	142.00	Smith Pamela	USA	2016
45–49	141.200	Kudo Mami	Japan	2011
50–54	132.202	Reutovitch Irina	Russia	2001
55–59	126,309	Esnaola Agesta Eva	Spain	2016
60–64	110.795	Muskett Valerie	New Zealand	2014
65–69	100.177	Lamothe Francoise	France	1989
70–74	103.600	Young Shirley	Australia	2002
75–79	71.508	Dinges Ursula	Germany	2017
80–84	48.995	Macklow Barbara	USA	2016
**24-h-run**				
World record	259.990	Bereznowska Patrycja	Poland	2017
35–39	250.621	Nagy Katalin	Hungary	2017
40–44	259.990	Bereznowska, Patrycja	Poland	2017
45–49	255.303	Kudo Mami	Japan	2011
50–54	243.657	Lomsky Sigrid	Germany	1993
55–59	217.811	Esnaola Agesta Eva	Spain	2016
60–64	195.975	Dahl Marianne	Germany	2004
65–69	176.519	Dahl Marianne	Germany	2009
70–74	176.461	Young Shirley	Australia	2002
75–79	123.543	Dinges Ursula	Germany	2017
80–84	91.233	Macklow Barbara	USA	2016
**48-h-run**				
World record	401.000	Bereznowska Patrycja	Poland	2019
35–39	369.749	Berces Edit	Hungary	2003
40–44	401.000	Bereznowska Patrycja	Poland	2019
45–49	368.687	Kudo Mami	Japan	2011
50–54	377.892	Trapp Sue	USA	1997
55–59	357.987	Eremina Galina	Russia	2009
60–64	305.82	Lamothe Francoise	France	1986
65–69	272.793	Lamothe Francoise	France	1991
70–74	239.763	Lamothe Francoise	France	1994
75–79	168.981	Biondic Erlinda	Canada	2017
80–84	162.187	Macklow Barbara	USA	2016
**6-days-run**				
World record	883.631	BarwickSandra	New Zealand	1990
35–39	806.000	Robinson Eleanor	Great Britain	1984
40–44	883.631	BarwickSandra	New Zealand	1990
45–49	773.976	David-Bodet Christine	France	2005
50–54	714.353	Nagyné Krisztina	Hungary	2013
55–59	653.457	Gielen Silke	Germany	2013
60–64	648.404	Lamothe Francoise	France	1985
65–69	552.890	Bayer Else	Germany	2004
70–74	600.290	Klein Helen	USA	1993
75–79	428.085	Biondic Erlinda	USA	2016

**Table 5:** World records in men in elite and age group athletes in time-limited races, from http://www.iau-ultramarathon.org/.

	**km**	**Name**	**Origin**	**Year**
**6-h-run**				
World record	97.200	Ritchie Donald	Great Britain	1978
35–39	97.200	Ritchie Donald	Great Britain	1978
40–44	89.973	Beneens André	Belgium	1996
45–49	87.579	Kruglikov Anatoly	Russia	2003
50–54	86.056	Lindemann Rainer	Germany	1999
55–59	83.490	Lantink Jan-Albert	Netherlands	2016
60–64	78.953	Schoonbroodt Jo	Netherlands	2012
65–69	74.756	Schoonbroodt Jo	Netherlands	2016
75–79	63.940	Gutbier Heinrich	Germany	2000
80–84	53.948	Simon Josef Mathias	Luxemburg	2015
**12-h-run**				
World record	163.785	Bitter Zach	USA	2013
35–39	163.785	Bitter Zach	USA	2013
40–44	161.800	Kouros Yiannis	Greece	1997
45–49	159.233	Kouros Yiannis	Greece	2001
50–54	142.924	Weir Denis	Great Britain	1990
55–59	131.128	Leighton Gard	USA	1989
60–64	132.565	McCorkindale Brian Robert	New Zealand	2015
65–69	122.752	McCorkindale Brian Robert	New Zealand	2017
70–74	109.600	Jones Max	Great Britain	1997
75–79	76.399	Vidan Melendez Ricardo	Spain	2016
80–84	89.732	Oliver Geoffrey	Great Britain	2013
85–89	60.963	Lightner Leo	USA	2013
**24-h-run**				
World record	303.506	Kouros Yiannis	Greece	1997
35–39	255.375	Rudolf, Tamas	Hungary	2017
40–44	303.506	Kouros Yiannis	Greece	1997
45–49	284.070	Kouros Yiannis	Greece	2002
50–54	266.596	Kouros Yiannis	Greece	2008
55–59	257.040	Kruglikov Anatoliy	Russia	2013
60–64	240.790	Courtillon Max	France	1990
65–69	217.532	Mainix Christian	France	2004
70–74	201.087	Vuillemenot, Roland	France	2016
75–79	178.898	Oliver Geoffrey	Great Britain	2009
80–84	152.295	Oliver Geoffrey	Great Britain	2013
**48-h-run**				
World record	473.495	Kouros Yiannis	Greece	1996
35–39	327.753	Mauduit Christian	France	2014
40–44	473.495	Kouros Yiannis	Greece	1996
45–49	443.337	Kouros Yiannis	Greece	2004
50–54	433.095	Kouros Yiannis	Greece	2008
55–59	425.125	Mainix Gilbert	France	1993
60–64	350.447	Boussiquet Jean-Gilles	France	2005
65–69	324.64	Mainix Christian	France	2004
70–74	253.497	Hauser Manfred	Germany	1998
75–79	246.242	Feller Horst	Germany	1999
80–84	202.535	Lardinois Robert	Belgium	1998
**6-days-run**				
World record	1036.8	Kouros Yiannis	Greece	2005
35–39	871.756	Mauduit Christian	France	2015
40–44	975.200	Boussiquet Jean-Gilles	France	1985
45–49	1036.800	Kouros Yiannis	Greece	2005
50–54	980.800	Mainix Gilbert	France	1986
55–59	1007.600	Mainix Gilbert	France	1992
60–64	841.600	Perdon George	Australia	1984
65–69	704.525	Audley George	Australia	2001
70–74	653.600	Young Cliff	Australia	1992
75–79	584.993	Kettle Drew	Australia	1995
80–84	487.631	Corbitt Ted	USA	2001

**Table 6:** World records in men and women in ultra-marathon running following IAU, from http://www.iau-ultramarathon.org/.

**Event**	**Time/Performance**	**Name and Origin**	**Year**
**Men**
50 km Road	2:43:38 h:min:s	Thompson Magawana (South Africa)	1988
50 km Track	2:48:06 h:min:s	Jeff Norman (Great Britain)	1980
100 km Road	6:13:33 h:min:s	Takahiro Sunada (Japan)	1998
100 km Track	6:10:20 h:min:s	Donald Ritchie (Great Britain)	1978
100 Miles Road	11:46:37 h:min:s	Yiannis Kouros (Greece)	1984
100 Miles Track	11:28:03 h:min:s	Oleg Kharitonov (Russia)	2002
100 Miles Indoor	12:56:13 h:min:s	Donald Ritchie (Great Britain)	1990
6 h Road	92.188 km	Tomasz Chawawko (Poland)	2004
6 h Track	97.200 km	Donald Ritchie (Great Britain)	1978
6 h Indoor	93.247 km	Denis Zhalybin (Russia)	2003
12 h Road	162.543 km	Yiannis Kouros (Greece)	1984
12 h Track	163.600 km	Zach Bitter (USA)	2013
12 h Indoor	146.296 km	Ryoichi Sekiya (Japan)	2007
24 h Road	290.221 km	Yiannis Kouros (Greece)	1998
24 h Track	303.506 km	Yiannis Kouros (Greece)	1997
24 h Indoor	257.576 km	Nikolai Safin (Russia)	1993
48 h Road	433.095 km	Yiannis Kouros (Greece)	1998
48 h Track	473.495 km	Yiannis Kouros (Greece)	1996
48 h Indoor	426.178 km	Tony Mangan (Ireland)	2007
**Women**			
50 km Road	3:08:39 h:min:s	Frith Van Der Merwe (South Africa)	1989
50 km Track	3:18:52 h:min:s	Carol Hunter-Rowe (Great Britain)	1996
100 km Road	6:33:11 h:min:s	Tomoe Abe (Japan)	2000
100 km Track	7:14:06 h:min:s	Norimi Sakurai (Japan)	2003
100 Miles Road	13:47:41 h:min:s	Ann Trason (USA)	1991
100 Miles Track	14:11:26 h:min:s	Pam Smith (USA)	2013
100 Miles Indoor	14:43:40 h:min:s	Eleanor Robinson (Great Britain)	1990
6 h Road	82.838 km	Ricarda Botzon (Germany)	2001
6 h Track	83.200 km	Norimi Sakurai (Japan)	2003
6 h Indoor	80.600 km	Marina Bychkova (Russia)	2003
12 h Road	144.840 km	Ann Trason (USA)	1991
12 h Track	147.600 km	Ann Trason (USA)	1991
12 h Indoor	135.799 km	Sumie Inagaki (Japan)	2007
24 h Road	252.205 km	Mami Kudo (Japan)	2013
24 h Track	255.303 km	Mami Kudo (Japan)	2011
24 h Indoor	240.631 km	Sumie Inagaki (Japan)	2011
48 h Road	368.687 km	Mami Kudo (Japan)	2011
48 h Track	397.103 km	Sumie Inagaki (Japan)	2010

## Are ultra-marathoners different to marathoners?

Generally, a long-distance runner competes for first time in an ultra-marathon after having completed several marathons. Most ultra-marathoners have run at least one marathon before the first start in an ultra-marathon and continue to participate in marathon races during their career as marathon runners (Knechtle, 2012).

Several studies compared training and anthropometric characteristics of marathoners and ultra-marathoners (Knechtle, 2012; Knechtle et al., 2012c; Rüst et al., 2012a,b) showing quite characteristic differences between the two groups of runners (Table 7). Ultra-marathoners have a higher number of successfully completed marathons than marathoners, whereas marathoners have a faster personal marathon race time than ultra-marathoners (Knechtle, 2012). Successful and experienced ultra-marathoners usually have several years of experience in ultra-marathon running (Knechtle, 2012).

**Table 7:** Comparison between marathon and ultra-marathon runners regarding anthropometric and training characteristics.

	**Marathon**	**Ultra-marathon**
Percent body fat	↑	↓
Skin fold thicknesses	↑	↓
Limb circumferences	↑	↓
Number of completed marathons	↓	↑
Personal best marathon time	↓	↑
Running kilometers	↓	↑
Running volume	↓	↑
Running speed during training	↑	↓

There are also differences in anthropometric characteristics between marathoners and ultra-marathoners (Knechtle et al., 2012c). Generally, ultra-marathoners appear to be thinner than marathoners and have thinner skin folds. Marathoners have a significantly lower calf circumference than ultra-marathoners, but thicker skin folds on the upper body (Rüst et al., 2012b). Ultra-marathoners have a smaller circumference on the upper arms and thighs and thinner skin folds on the upper body than marathoners (Rüst et al., 2012a). Ultra-marathoners have thicker calves, but are thinner in the upper body than marathoners, probably due to adaptation due to the many training km and the many ultra-marathons.

Ultra-marathoners also show differences in training compared to marathoners. This is especially evident in the scope and intensity of running training. Ultra-marathoners run much slower in training than marathoners (Knechtle, 2012; Knechtle et al., 2012c), but complete more running kilometers and more running hours per week in training (Rüst et al., 2012b). One reason that ultra-marathoners train and compete longer distances than marathoners may be the fact that ultra-marathoners have a higher pain tolerance compared to other persons (Freund et al., 2013). Runners with increasing length in their race seem to invest more and more in training and are getting thinner and thinner.

## What distinguishes the successful ultra-marathoner?

Key predictors of a successful ultra-marathon finish were age (Knechtle et al., 2010b; Rüst et al., 2012b), specific aspects of anthropometry such as low body fat (Knechtle et al., 2012c), low BMI (Hoffman, 2008), and low limb circumferences (Knechtle et al., 2008b). Other aspects included fast personal best running times and extensive previous race experience (Knechtle et al., 2009, 2011d), and a high running speed and a high running volume during training (Knechtle et al., 2010a, 2012c; Rüst et al., 2012b).

Depending on the kind of the analysis (i.e., correlation of individual variables with running performance or inclusion of multiple variables in a regression model), results were different. For example, the thigh skinfold thickness as a highly predictive parameter for shorter running distance races (Arrese and Ostariz, 2006) is only significant in a single correlation for ultra-marathoners, but not in a multi-variate regression analysis involving multiple variables (Knechtle et al., 2009, 2011e). Analyzing the relationship between variables of anthropometric characteristics and race performance, the percentage of body fat and BMI appear to be the most important variables (Hoffman, 2008; Hoffman et al., 2010a). In 100-mile ultra-marathoners, a direct linear relationship between a low percentage of body fat and fast race times were found (Hoffman, 2008). In the same group of runners, faster ultra-marathoners and successful finishers had a lower body fat percentage than slower ultra-marathoners and non-finishers (Hoffman et al., 2010a).

When various anthropometric characteristics such as skeletal muscle mass, body fat and running training were examined in multi-variate analyses, only low body fat and fast running speed in training were correlated with fast race times (Knechtle et al., 2012c). In ultra-marathoners, weekly running kilometers and average running speed during training were negatively and the sum of the skin folds positively correlated with race times (Knechtle et al., 2010a).

Apart from aspects of anthropometry and training, age also seems to have a major impact on ultra-marathon performance. In 100-km ultra-marathoners, age, BMI and body fat were positively and the weekly running kilometers negatively correlated with race time (Rüst et al., 2012b). Low body fat and a fast running speed during training are the most important predictors for a fast ultra-marathon race time.

Besides these variables, experience seems to be the most important variable for a successful performance in an ultra-marathon (Knechtle et al., 2009, 2010a, 2011c). Ultra-marathoners need several years to reach their fastest running speed in a competition (Rae et al., 2005). The personal marathon best time was an important predictor variable for mountain ultra-marathoners (Knechtle et al., 2010a). In 24-h ultra-marathoners, aspects such as anthropometric characteristics and training running volume showed no correlation with race performance, and the personal marathon best time showed the highest impact on ultra-marathon race performance (Knechtle et al., 2009). To reach a maximum of km in a 24-h ultra-marathon, ultra-marathoners should have a personal best marathon time of 3:20 h:min and have completed a continuous training run of at least 60 km before the race, while anthropometric aspects such a low body fat or thin skin folds showed no relationship with race performance (Knechtle et al., 2011c). Therefore, apart from low body fat and fast running speed during training, previous experience such as a fast personal best marathon time seem to be the most important predictors for a fast ultra-marathon race time.

Other aspects were also investigated where cognitive functions should not be ignored. Faster ultra-marathoners seem to focus on the relevant, unlike slower ultra-marathoners (Cona et al., 2015). Also important are physiological variables such as maximal oxygen uptake (VO_2_max) (Davies and Thompson, 1979). In addition, faster runners experienced less pain in a 100-mile ultra-marathon, in the sense that these ultra-marathoners had a better exercise-induced analgesia (Hoffman et al., 2007). Another aspect is the pacing, or how ultra-marathoners distribute their energy across the race (Micklewright et al., 2015). In the 100-mile “Western States Endurance Run,” the winners initially ran behind the leaders during the race. In the middle of the race, they took over the lead themselves and the top ultra-marathoners were consistent over the entire race distance (Hoffman, 2014). In 24-h ultra-marathoners, the fastest runners start at lower relative intensities and display a more even pacing strategy than slower runners (Bossi et al., 2017). In 100-km ultra-marathon running, the strategy seems different. Fast 100-km ultra-marathoners tackle the race at a high pace, keeping the pace constant for a long time, and do not make any tempo changes (Lambert et al., 2004). Successful elite ultra-marathoners make little to no breaks during the race (Kerhervé et al., 2015) and often run in groups of runners of the same speed (Tan et al., 2016). Less experienced ultra-marathoners are advised to start at a rather low initial pace that they are most likely to endure during the full distance (Tan et al., 2016). Pacing has also been studied in a few studies for age group runners. It was shown the older runners during a 100-km ultra-marathon did not slow down as younger runners and that especially the young runners in the age group 18–24 years were slower than all other age group runners (Rüst et al., 2015). Taken together, pacing in an ultra-marathon is crucial to finish among the top.

There are also differences between finishers and non-finishers in strategy during the race. During an ultra-marathon many different problems occur, such as muscular spasms, overuse injuries, digestive problems, motivation problems and sleep deprivation (Hurdiel et al., 2015). Experienced ultra-marathoners also have less medical problems such as muscle cramps, digestive problems, etc. (Schwabe et al., 2014). Successful finishers tackle the competition in small stages, paying attention to running speed, nutrition, hydration and team support (Holt et al., 2014). A successful ultra-marathon finish is a good life experience while a non-finish is a big disappointment (Holt et al., 2014). Motivational self-talk seems to be an effective tool to cope with exertion, as well as other stressors such as blister discomfort and adverse conditions (McCormick et al., 2015).

The management of sleep deprivation seems to be central in long to very long ultra-marathons (Van Helder and Radoki, 1989). It has been shown in the “North-Face Ultra-Trail du Mont-Blanc” (UTMB) that ultra-marathoners who finished without sleep were faster than ultra-marathoners with sleep breaks. Runners who adopted a sleep management strategy based on increased sleep time before the race completed the race faster (Poussel et al., 2015).

## Women in the ultra-marathon

The proportion of women in ultra-marathons was very low at the beginning of the ultra-running movement. In 100-mile ultra-marathons held in the United States, the proportion of women in the late 1970s increased from almost no participant to ~20% since 2004 (Hoffman et al., 2010b). This percentage has remained fairly stable at ~10–20% in recent years (Hoffman et al., 2010b; Eichenberger et al., 2012; Fonseca-Engelhardt et al., 2013). In most ultra-marathons, the proportion of women has increased in recent years such as in the “Badwater” to ~19.1% and in the “Spartathlon” to ~12.5% (Fonseca-Engelhardt et al., 2013), and in the “Swiss Alpine Marathon” held in Switzerland to ~16% (Eichenberger et al., 2012).

The rather low participation of women can have different reasons. One important reason is the different motivation of women toward men in ultra-marathon running. While female ultra-marathoners tend to be more intrinsically motivated (Krouse et al., 2011), less concerned with the competitive nature (Frick, 2011) and more attentive to health (Krouse et al., 2011), men have a competitive nature in which they want to compete with opponents and win a race (Doppelmayr and Molkenthin, 2004). Women also have a higher flow experience during an ultra-marathon than men (Wollseiffen et al., 2016).

An ealier assumption was that women become less tired than men during an ultra-marathon (Bam et al., 1997). Even though women run faster in exceptional cases than men, men are always faster than women in ultra-marathons (Coast et al., 2004; Eichenberger et al., 2012; Peter et al., 2014). Coast et al. (2004) compared the world's best performance for running distances from 100 m to 200 km, showing that men were on average ~12.4% faster than women.

It also known that the difference between the sexes increased with increasing race distance or race duration (Coast et al., 2004). An analysis of all 24-h ultra-marathons held worldwide between 1977 and 2012 showed that men were on average ~5% faster than women when all runners were considered (Peter et al., 2014). When the top 10 were evaluated, the difference was ~13%, for the top 100 the difference was on average ~12%, and when the fastest were taken per calendar year, the difference was again ~13% (Peter et al., 2014). Women seem to aim for a different running tactic than men in the ultra-marathon. In a 100-km ultra-marathon during the Masters World Championships, women were relatively slower than men at the start of the race, but at the end of the race they had a higher running speed than men (Renfree et al., 2016). However, in recent years women have been able to reduce the gap of men (Eichenberger et al., 2012; Rüst et al., 2013b; Peter et al., 2014). An analysis of 24 h ultra-marathoners showed that the gap fell to ~17% in the annual fastest, to ~11% for the annual 10 fastest, and to ~14% in the annual 100 fastest (Peter et al., 2014). An analysis of all 100-mile ultra-marathons in the world from 1998 to 2011 showed that women were able to close the gap to men to ~14% (Rüst et al., 2013b).

In recent decades, women have been able to reduce the gap to men, especially in the age group categories in which relatively many women were at the start (Knechtle et al., 2016b). The difference in performance between the sexes is influenced by several variables. Thus, the difference between the sexes is greatest when fewer women than men participate, especially on the shorter ultra-marathon distances (Senefeld et al., 2016). Considering the trend over different running distances in the last decades, it has been shown that over longer running distances such as 200 and 1,000 km the difference between the sexes remained the same. On the other hand, women approached to men over shorter running distances of 50 and 100 km. Since this trend is non-linear, it is absolutely unlikely that women will approach men in the near and far future even further and they will never beat the men (Zingg et al., 2014a). In rare cases, however, women may be able to beat all men in an ultra-marathon (www.runnersworld.com/trail-running-training/why-women-rule-ultrarunning). For example, the Japanese Hiroko Okiyama was able to beat all men in the “Germanyrun” 2007 (Knechtle et al., 2008a). Future studies need to investigate whether women were able to reduce the gap to men in older age groups.

## Age of peak performance

**Figure 2:**
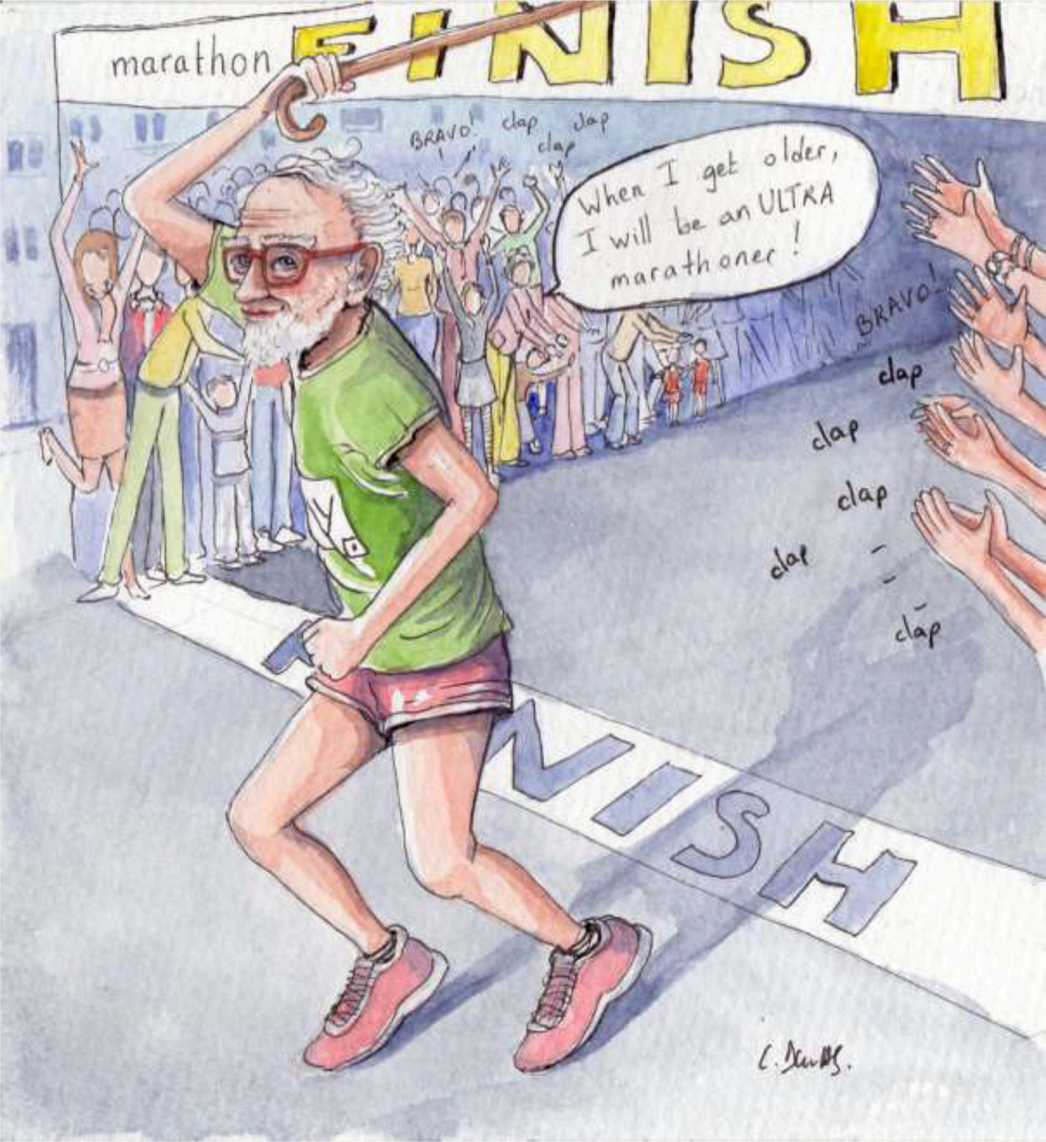
Ultra-marathoners are older than marathoners. Figure by Céline Dewas.

The age of the best ultra-marathon performance has been analyzed in several studies in recent years (Eichenberger et al., 2012; Knechtle et al., 2012d; Fonseca-Engelhardt et al., 2013; Rüst et al., 2013b; Zingg et al., 2013b; Peter et al., 2014; Cejka et al., 2015; Nikolaidis and Knechtle, 2018b). In general, the best ultra-marathon performance is achieved at an older age than the best performance over half-marathon and marathon (Figure 2; Knechtle et al., 2012d, 2014b; Romer et al., 2014; Rüst et al., 2014; Zingg et al., 2014b; Nikolaidis and Knechtle, 2018a,b). The best marathon race time is achieved at the age of ~30 years, where performance level and nationality are important predictor variables of the age of peak performance (Hunter et al., 2011; Knechtle et al., 2014a, 2016a; Lara et al., 2014; Nikolaidis et al., 2016). In ultra-marathons, the age of best performance is often ~35 years or older (Knechtle et al., 2014b; Rüst et al., 2014; Knechtle and Nikolaidis, 2017; Nikolaidis and Knechtle, 2018a,b).

It seems that the age of peak ultra-marathon performance increases with increasing length of the race distance. In 50-km ultra-marathon running, the age of the best performance is at ~39–40 years (Nikolaidis and Knechtle, 2018a). In 100-km ultra-marathon running, the best race times are achieved at the age of 30–50 years for men and 30–55 years for women (Knechtle et al., 2012d). In 100-mile ultra-marathon running (161 km), the best race times were achieved at the age of 30–39 years for men and 40–49 years for women (Hoffman, 2010). The age of the best performance in ultra-marathons has increased in recent decades, with an increase especially in the long to very long ultra-marathons and in those runners who had finished many to very many ultra-marathons (Knechtle et al., 2014b). The older age of peak performance in the ultra-marathon comparted to marathon might be explained from the observation that ultra-marathon runners have finished many marathon races before finishing an ultra-marathon.

With regards to sex differences in the age of peak performance, women achieve their best ultra-marathon race time at the same age as men (Knechtle et al., 2012d; Rüst et al., 2013a; Zingg et al., 2013b; Peter et al., 2014; Cejka et al., 2015). In 100-km ultra-marathon running, the age of the fastest annual female and male finishers was ~35 years for both sexes (Cejka et al., 2015). In 24-h ultra-marathon running (Zingg et al., 2013b) as well as in “Badwater” and “Spartathlon” (Zingg et al., 2013a), the best performance was achieved for both sexes at the age of ~40 years.

Considering newer studies with more runners and longer time frames, there seems to be a difference in the age of peak performance between women and men depending upon the length of the performance. In 50-km ultra-marathons, women seemed to achieve the best race time later in life compared to men (Nikolaidis and Knechtle, 2018a). In 100-km ultra-marathons, however, the age of peak performance was younger in women than in men (Nikolaidis and Knechtle, 2018b). These disparate findings were most likely explained by the different ages of women and men competing in these race distances. Furthermore, the approach of the analysis seems of importance. When the fastest runners in the “Comrades Marathon” were considered in 1-year intervals, the fastest running speed was achieved in men at the age of 36.38 years. For the fastest women, the age of the fastest running speed was at the age of 32.75 years. When all runners were considered, men achieved the best ultramarathon performance ~6 years earlier than women, whereas when the fastest runners were considered, men achieved the best performance ~4 years later than women (Knechtle and Nikolaidis, 2017).

It seems that the age of peak performance on the long running distances increases with increasing distance and/or race duration (Rüst et al., 2013b; Peter et al., 2014). In 100-miles ultra-marathons, the age of the fastest men was at ~37 years and the fastest women at ~39 years (Rüst et al., 2013b). In 24-h ultra-marathoners who reached 200 km and more, the fastest men are ~44 years and the fastest women ~43 years old (Peter et al., 2014). Table 8 summarizes the age of best performance in the time-limited ultra-marathons of 6 h to 10 days (Knechtle et al., 2014b). While the youngest age of ~37 years was found in women over 10 days, the age of best performance in men increases with increasing duration. Thus, the age of ~35 years in 6 h and ~37 years in 12 h increases to ~48 years in 72 h, 6 and 10 days (Rüst et al., 2014). However, future studies need to confirm these findings in distance-limited ultra-marathons such as races held in kilometers from 50 to 1,000 km and races held in miles from 50 to 3,100 miles.

**Table 8:** Increase in age of peak performance with increasing duration in ultra-marathon running.

**Duration**	**Age (mean and 95% CI)**
6-h run	33.7 years (32.5–34.9)
12-h run	39.4 years (38.9–39.9)
24-h run	43.5 years (43.1–43.9)
48-h run	46.8 years (46.1–47.5)
72-h run	43.6 years (40.9–46.3)
6-days run	44.8 years (43.9–45.7)
10-days run	44.6 years (42.9–46.3)

When investigating the age of the best ultra-marathon performance over the years, in some cases the age of the year's fastest finishers increased (Eichenberger et al., 2012; Rüst et al., 2013b; Peter et al., 2014) or decreased (Fonseca-Engelhardt et al., 2013) over time. For example, the age of the fastest men in the 24-h ultra-marathons rose from 23 years in 1977 to 53 years in 2012 (Peter et al., 2014). These disparate findings are most likely due to the different length or durations of the races and the different time frames.

In addition to elite ultra-marathoners, the number of age-group runners has increased and their performance has improved (Jampen et al., 2013; Rüst et al., 2013a). In the “Swiss Alpine Marathon,” the number of runners older than 30 years increased significantly and the performance of women in age group 40–44 years significantly improved (Rüst et al., 2013a). In the “Marathon des Sables,” the number of finishers older than 40 years increased and the performance of men in the age groups 35–39 and 40–44 years improved (Jampen et al., 2013). The trend is different for the different age categories. Considering the best 100-km and 100-mile ultra-marathoners, younger runners (i.e., 25–35 years old) and older runners (i.e., 50–65 years old) can still improve their performance in the next years and decades (Rüst et al., 2014). A possible explanation for the relatively old age of ultra-marathoners could be the fact that the mean age at the first start of an ultra-marathon is quite high. Hoffman and Krishnan (Hoffman and Krishnan, 2013) showed that the age at the start of the first ultra-marathon was ~36 years.

## Energy and fluid metabolism

The homeostatic control of energy balance, i.e., the optimization of the relationship between energy intake and expenditure, is a major concern in ultra-marathon due to its large energetic demands. Two important problems regarding energy and fluid metabolism in ultra-marathon running are the energy deficit and a potential fluid overload leading to exercise-associated hyponatremia.

## Energetic requirements in ultra-marathon running

An ultra-marathon leads to a great energetic demand (Thompson et al., 1982; Stuempfle et al., 2011). In a 24-h ultra-marathon, runners were only able to maintain their running speed for the first 6 h, after which they slowed continuously down (Gimenez et al., 2013). It could be shown that a runner consumed about 6,300 kcal per day during his “Run around Australia” (Hill and Davies, 2001). In order to cover the energy consumption, the runners have to consume high amounts of energy during an ultra-marathon. Successful ultra-marathon finishers are more likely to consume and deliver the required energy during performance than non-finishers (Glace et al., 2002).

In general, ultra-marathoners are unable to meet their energy demands during a race by feeding them (Machefer et al., 2007; Enqvist et al., 2010) and in some cases a considerable energy deficit arises (O'Hara et al., 1977; Hill and Davies, 2001; Enqvist et al., 2010). The energy deficit, i.e., negative energy balance, is due to insufficient energy intake, most likely due to suppression of appetite and digestive problems during the race (Enqvist et al., 2010). The negative energy balance reduces the concentration of leptin in the serum after a long endurance performance (Landt et al., 1997). In turn, decreased levels of leptin might be a concern for overtrained endurance runners (Bobbert et al., 2012). The abovementioned studies focused on the quantification of energy balance rather than to identify the energy sources for a race.

Nevertheless, knowledge about the main energy sources for an ultramarathon race—not only in terms of calories, but also in terms of choice of carbohydrates, lipids and protein—is necessary in order to develop optimal nutritional strategies. Carbohydrates are generally the most important source of energy during an ultra-marathon (Eden and Abernethy, 1994; Case et al., 1995; Fallon et al., 1998). A total of 88.6% of the energy in 100-km ultra-marathon comes from the intake of carbohydrates, only 6.7% from the intake of fat and 4.7% from the intake of protein (Fallon et al., 1998). However, ultra-marathoners generally do not meet the required intake of carbohydrates (Martinez et al., 2017). However, carbohydrate intake—regardless of type or amount—can not delay glycogen breakdown during performance (Noakes et al., 1988). In addition, top runners are able to maintain a high running speed—independent of the energy supply—without a decrease on plasma glucose concentration during performance (Sengoku et al., 2015). For longer ultra-marathons, the proportion of fat and protein to energy contribution seems to increase. In ultra-marathoners in a race over 1,005 km in 9 days, the average daily intake of carbohydrates dropped to 62%, while the proportion of fat increased to 27% and of protein to 11% (Eden and Abernethy, 1994). A recent study showed that the intake of fat increased with increasing race distance (Martinez et al., 2017). Thus, it was observed that the ratio among carbohydrates, lipids and protein follows the current nutritional recommendations for marathon runners (Stellingwerff, 2013) with the contribution of carbohydrates and lipids decreasing and increasing, respectively, as the distance of ultramarathon increases.

The result of the energy deficit is a decrease in body mass (Belli et al., 2016), where both fat-free body mass (i.e., muscle mass) and fat mass are reduced (Knechtle et al., 2011g; Schütz et al., 2013). The use of fat as an energy substrate is shown by the increase in free fatty acids during an ultra-marathon (Pestell et al., 1993; Fallon et al., 1999a). Another problem of the energy deficit is the insufficient intake of vitamins (Holtzhausen and Noakes, 1995; Machefer et al., 2007) which is preferably seen in the pre-race preparation. Overall, ultra-marathoners in very long races without brakes will face an energy deficit with a decrease in solid mass. To date, there is no obvious solution to solve this problem.

## Exercise-associated hyponatremia in ultra-marathon running

During an ultra-marathon, all runners experience dehydration with a decrease in body mass (Holtzhausen and Noakes, 1995). The greatest body weight loss occurrs in the first hours of an ultra-marathon (Kao et al., 2008). Therefore, ultra-marathoners have to consume large amounts of fluids during a race to avoid dehydration in terms of body weight loss (Newmark et al., 1991).

However, consumption of large amounts of fluid can lead to dilutional hyponatremia or exercise-associated hyponatremia hyponatraemia (Barr and Costill, 1989; Eijsvogels et al., 2008; Bürge et al., 2011; Knechtle et al., 2012b; Chlibkova et al., 2014). The association between excessive water intake and exercise-associated hyponatraemia was first recognized in 1981 in a runner competing in the “Comrades Marathon” (Noakes, 2008). Symptomatic exercise-associated hyponatraemia is described not only in ultra-marathoners (Hew-Butler et al., 2007) but also in marathoners (Spormann et al., 2012; Wellershoff, 2013).

Exercise-associated hyponatremia is defined as a plasma sodium level of 135 mmol/l or lower (McGreal et al., 2016). Fluid overload can be seen not only from the change in electrolytes, but also from changes in plasma volume (De Paz et al., 1995; Costa et al., 2014), total albumin, and albumin (Fallon et al., 1999a).

In ultra-marathon running, there is no need to drink more fluid than required, or to over-drink (Knechtle et al., 2012e). Even if ultra-marathoners lose more than 3% of their body weight, there is no reason to over-drink to prevent overheating (Valentino et al., 2016). In an ultra-marathon, a runner of ~70 kg body mass must lose ~1.9–5.0% of body mass to maintain the water supporting body water balance while also avoiding overhydration (Hoffman et al., 2017b). A recent study showed that water intake decreased with increasing race distance (Martinez et al., 2017).

In general, ultra-marathoners do not seem to consume excessive fluid (Knechtle et al., 2011b) and an ultra-marathoner should not experience any fluid overload (Knechtle et al., 2011f). In a 100-km ultra-marathon, faster ultra-marathoners drank more liquid than slower ultra-marathoners and faster ultra-marathoners lost more body weight than slower ultra-marathoners. The weight loss was greater with less fluid intake (Knechtle et al., 2012e). Due to the fact that faster ultra-marathoners lost more body weight, the weight loss can be ergogenic and the ultra-marathoners achieve a faster race time due to their lower body weight (Knechtle et al., 2012e). In a 100-mile ultra-marathon, increased body weight losses did not lead to a limited performance but were rather performance-enhancing (Landman et al., 2012). In a 100-km ultra-marathon, runners with a greater body weight loss were faster (Rüst et al., 2012c).

Ultra-marathoners also seem to be able to self-regulate their plasma sodium during an ultra-marathon. In an ultra-marathon, the prevalence of exercise-associated hyponatremia was considerably higher during the race than after the race (Cairns and Hew-Butler, 2015). It has also been reported that ultra-marathoners with the highest fluid intake had the least hemodilution without changes in plasma sodium and potassium (Kaminsky and Paul, 1991). Often, exercise-associated hyponatremia is clinically not perceivable, in very few cases; however, it can lead to major medical problems. Thus, a case of a 57-year-old man who developed exercise-associated hyponatremia during a 100-mile ultra-marathon is described. Hyponatraemia resulted in rapid neurological deterioration and cardiovascular instability (Surgenor and Uphold, 1994).

Exercise-associated hyponatraemia is a relatively frequently detectable electrolyte disorder in ultra-marathoners (Noakes and Carter, 1982; Knechtle et al., 2011b; Hoffman et al., 2012b; Costa et al., 2013), whereby high ambient temperatures must be given a great importance (Lebus et al., 2010; Costa et al., 2013). The prevalence of exercise-associated hyponatremia in a 225-km ultra-marathon over five stages was ~42% with ambient temperatures as high as ~40°C (Costa et al., 2013). In the “Rio de Lago 100-Mile Endurance Run,” held in 2008 in Granite Bay, California, USA, the prevalence of exercise-associated hyponatraemia was ~51.2% (Lebus et al., 2010). However, in 100-mile ultra-marathoners, the prevalence of exercise-associated hyponatraemia may be as low as ~30% (Hoffman et al., 2012b). A great cold seems also to be a major risk factor for exercise-associated hyponatremia. In a 100-mile ultra-marathon held in Alaska, 44% of runners had exercise-associated hyponatraemia (Stuempfle et al., 2002).

In ultra-marathons held in temperate climates, exercise-associated hyponatraemia is relatively uncommon (Cuthill et al., 2009; Bürge et al., 2011; Knechtle et al., 2011c,f, 2012b). In ultra-marathons held in Switzerland, Europe, a low prevalence of exercise-associated hyponatraemia was found. In the “Swiss Jura Marathon,” a mountain ultra-marathon over 350 km in 7 stages at medium to low temperatures held in the Swiss Jura region from Geneva to Basel, the prevalence of exercise-associated hyponatraemia was ~8% (Knechtle et al., 2012b). In the “100 km Lauf Biel” (Bürge et al., 2011; Knechtle et al., 2011f) and at the “24 h Run Basel” at medium to low temperatures, no case of exercise-associated hyponatraemia could even be detected (Knechtle et al., 2010a).

Also, the country in which the ultra-marathons take place seems to be of importance. The prevalence of exercise-associated hyponatraemia in ultra-marathons held in the USA is higher than in ultra-marathons held in Europe. While the prevalence of exercise-associated hyponatremia was ~30% in the “Western States Endurance Run” held in California (Hoffman et al., 2012b), it was no more than ~8% in ultra-marathons held in Switzerland (Knechtle et al., 2010a, 2012b; Bürge et al., 2011). Even in ultra-marathons held in the Czech Republic, the prevalence of exercise-associated hyponatraemia was very low (Chlibkova et al., 2014) as was the case in Asia, where the prevalence of exercise-associated hyponatremia was only ~3% in an 84-km ultra-marathon (Lee et al., 2011). In Australia, no case of exercise-associated hyponatremia could be detected in an ultra-marathon (Reid and King, 2007). The USA may be a special case, since a lot of drinking is promoted there and body weight loss can lead to disqualification. For example, in the “Vermont's 100-mile” race, athletes are required to: “Runners may be asked to weigh in or undergo further evaluation” and “7% of pre-race weight wants to be allowed to continue.”

The prevalence of exercise-associated hyponatraemia increased in a multi-stage ultra-marathon with increasing number of stages. In a 250-km multi-stage ultra-marathon, the prevalence of exercise-associated hyponatraemia per stage was 1.6% (S1), 4.8% (S3), and 10.1% (S5) with a cumulative incidence of 14.8% (Krabak et al., 2017).

It has already been investigated whether exercise-associated hyponatraemia can be prevented by means of education, drinking behavior or sodium intake. However, such efforts can not influence the onset of exercise-associated hyponatraemia (Winger et al., 2013). Several studies investigated whether the intake of sodium during an ultra-marathon is important (Hoffman and Stuempfle, 2014, 2016b). Considering 100-mile ultra-marathoners in the USA, up to 100% of runners consume supplements with sodium (Hoffman and Stuempfle, 2014). The benefits of this intake, however, seem to be very limited. Studies of 100-mile ultra-marathoners showed that supplemental intake of sodium during a heat-run did not affect the runners' state of hydration (Hoffman and Stuempfle, 2014, 2016b).

Very recent investigation showed an association between CK-values and exercise-associated hyponatremia. One study compared runners with and without exercise-associated hyponatremia on the same 100-mile ultra-marathon. Runners with exercise-associated hyponatraemia had less experience over 100 miles and a higher CK. In addition, there was an inverse relationship between plasma sodium levels and CK after arrival. On the other hand, there were no differences in age, sex, weekly training kilometers, use of sodium supplements during exercise, body weight loss, frequency of water intake, ingestion of pain killers, and symptoms during the race (Hoffman et al., 2013a). Whether there is really a correlation between CK-values after an ultra-marathon and exercise-associated hyponatraemia is still uncertain (Hoffman et al., 2012a).

**Figure 3:**
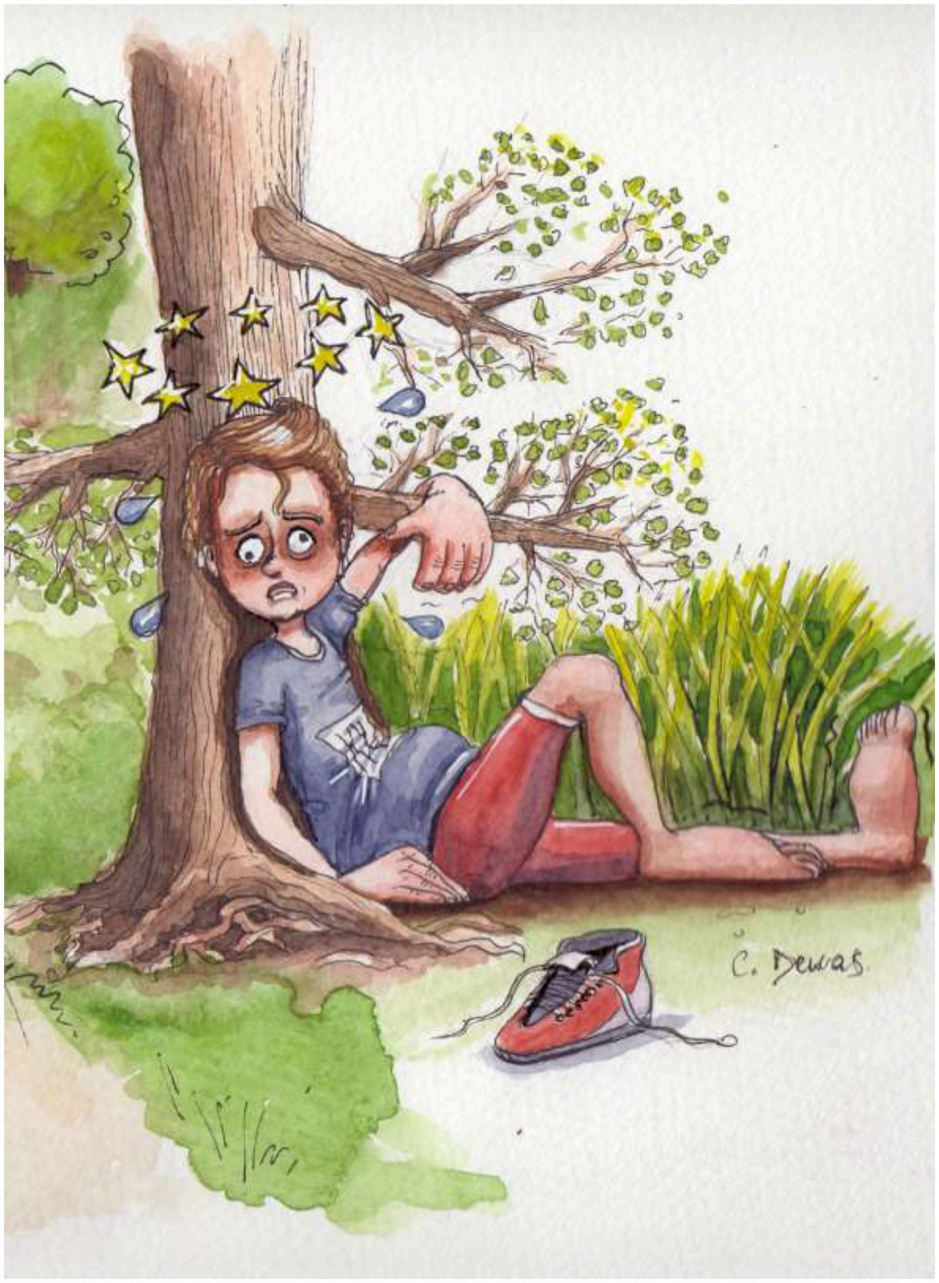
Fluid overload can result in exercise-associated hyponatremia, limb swelling and exercise-associated hyponatremic encephalopathy. Figure by Céline Dewas.

Excessive fluid overload, in addition to biochemically detectable hyponatraemia, can lead to hyponatremic encephalopathy and swelling of the hands and feet (Figure 3). Fluid overload can lead to exercise-associated hyponatremic encephalopathy with severe consquences (Frizzell et al., 1986). Several cases of long-distance runners with symptomatic exercise-associated hyponatraemia with altered behavior, seizures and edema are known (Bruso et al., 2010; Hoffman and Myers, 2015; Pearce et al., 2015). Excessive hydration during exercise also has negative effects on the volume of the feet. Recent studies have shown an association between fluid intake during an ultra-marathon and feet swelling during running (Bracher et al., 2012; Cejka et al., 2012). Recent work also pointed to an association between exercise-associated hyponatraemia and skeletal muscle damage. A 24-h ultra-marathon revealed that hyponatremia preceded the increase in CK (Cairns and Hew-Butler, 2016).

Surprisingly, however, hypernatremia is much more common than hyponatraemia. In 133 collapsed runners in the “Comrades Marathon,” ~45% of runners had hypernatremia, ~2% hyponatremia and ~53% normonatremia (Hew-Butler et al., 2007). However, another study on the “Comrades Marathon” found hyponatremia in ~9% of the collapsed runners (Noakes et al., 1990). It must be noted that collapsed runners do not always have an electrolyte imbalance. In most cases, runners collapse to the target line and have an electrolyte imbalance in only ~16% of the cases, as opposed to ~19% for non-collapsed runners (Holtzhausen et al., 1994).

Some of these ultra-marathons take place in difficult external conditions such as extreme heat (Knoth et al., 2012). A problem of the heat is the fact that the performance is partly considerably impaired (Parise and Hoffman, 2011; Wegelin and Hoffman, 2011). A run in the heat like “Badwater” in Death Valley in the USA inevitably leads to a rise in body core temperature. However, the increase is dependent on running speed, with a slight increase in the fastest runners (Brown and Connolly, 2015). In addition, running in the sun seems to significantly increase the risk of malignant skin tumors (Ambros-Rudolph et al., 2006).

A sufficient adaptation to the heat can be achieved with adequate acclimatization (Sandström et al., 2008; Costa et al., 2013). Four days of short term heat acclimation facilitates effective perceptual adaptations (Willmott et al., 2017). The adaptation to the heat can also be achieved by pauses during heat or tropical runs (Joslin et al., 2015). It is also important that an appropriate cooling is initiated immediately in case of suspected overheating or heat stroke (Rae et al., 2008). High heat can also lead to a nutritional problem. For example, a study on the “Marathon des Sables” showed that runners had major nutritional problems due to the dry mouth and inability to consume highly sugared energy gels and sports drinks when the products were heated due to the ambient heat (McCubbin et al., 2016). Finally, ultra-marathoners will not develop exercise-associated hyponatremia when they drink *ad libitum* with no fluid overload. Athletes must be aware that extreme weather conditions such as extreme heat and extreme cold might lead to fluid overconsumption.

## Damage to tissues and organs

Ultra-marathon running leads to selective damages in different tissues and organs. The most apparent problem is muscular pain. However, a negative effect can be found for the heart, the liver, the kidneys, the bone, the digestive tract and both the immune and the hormone system.

## Musculoskeletal problems in ultra-marathoners

Training for an ultra-marathon and ultra-marathons *per se* usually result in a damage to the musculo-skeletal system (Khodaee et al., 2015; Scheer and Murray, 2015), with the extent of damage depending on the length of the ultra-marathon (Kim et al., 2009). The damage is generally only of minor importance (Vernillo et al., 2016) and beginners are more often affected than experienced ultra-marathoners (Videbæk et al., 2015). Running over long to very long distances can cause minor damages such as muscle soreness on the musculature, but can also lead to substantial problems in the joints and tendons (Fallon, 1996; Freund et al., 2012; Lopes et al., 2012). In longer ultra-marathons, ~50–60% of participants experience musculoskeletal problems (Hutson, 1984). However, it is also possible in rare cases to cross a continent without any injury (Lathan and Cantwell, 1981). Various studies using Magnetic Resonance Imaging (MRI) have shown damages such as fluid retention around tendons, tissue edema, and damage to cartilage (Theysohn et al., 2013). Such overuse injuries are the most common reason that ultra-marathoners have to interrupt training, while other reasons such as work or family never lead to a training interruption (Hoffman and Krishnan, 2013).

The most common injuries in ultra-marathoners involve the lower limb, such as the ankle and the knee (Fallon, 1996; Scheer and Murray, 2011; Hetsroni and Mann, 2012), and are manifested as Achilles tendon inflammation and femoropatellar syndrome (Lopes et al., 2012). When a knee is already damaged, a further ultra-marathon has a further negative effect on the joint (Hagemann et al., 2008). At the lower leg, shin splints (i.e., medial tibial stress syndrome) occur frequently. In the ankle, inflammation of the extensor tendons is common. The pattern of overuse injuries depends on the kind of running. While ankle problems are more likely to occur in trail running, knee problems are more common in road running (Bishop and Fallon, 1999).

The symptoms are often relatively small, even in longer multi-stage ultra-marathons (Fallon, 1996; Krabak et al., 2011). During a multi-stage ultra-marathon of 5 days and covering 219 km, ~22% of the runners had lower limb problems, mainly affecting the knee (Scheer and Murray, 2011). In an ultra-marathon of 1,005 km from Sydney to Melbourne, 32 runners experienced a total of 64 different problems, of which the knee (~31.3%) and the ankle (~28.1%) were the most frequently affected joints (Fallon, 1996). Achilles tendonitis, femoropatellar pain, and inflammation of extensor tendons in the foot were the most common problems in a 6-day ultra-marathon (Bishop and Fallon, 1999).

For very long ultra-marathons, such as crossing a continent, adaptations of lower limb tissues occur. An increase in the diameter of the Achilles tendon was demonstrated during the “Trans Europe Footrace,” a multi-stage ultra-marathon covering 4,487 km from Bari in Italy to the North Cape. This thickening of the tendon was interpreted as an adaptation to the stress of the ultra-marathon (Freund et al., 2012). However, intraosseous changes and subcutaneous edema were also detected. Changes of this kind occurred rather in those runners who had to stop the race early (Freund et al., 2012).

Blisters on the feet are also quite common in ultra-marathon running (Scheer et al., 2014). However, it has been shown that the development of blisters is dependent on the experience of the ultra-marathoner. With increasing experience in ultra-marathon running, there are fewer blisters on the feet (Scheer et al., 2014).

Despite all problems with overuse injuries in ultra-marmathon running, a long run also seems to have a favorable influence on the cartilage (Mündermann et al., 2017). Runners in the “Trans Europe Footrace” initially showed a damage of the ankle cartilage which could regenerate during the the race (Schütz et al., 2014). MR-findings showed that elevated T2-values recovered during the second half of the “Trans Europe Footrace” supported the evidence that this response is a physiological adaptive mechanism of chondrocyte function via upregulation of *de novo* synthesis of proteoglycans and collagen (Schütz et al., 2014). In addition to MR-findings, changes in the concentrations of cartilage biomarkers in the serum showed that articular cartilage is able to adapt during a multi-stage ultra-marathon such as the “Trans Europe Footrace” (Mündermann et al., 2017). Although overuse injuries of the lower limbs may be frequent, not all ultra-marathoners must suffer from this problem. Most likely very experienced and highly trained athletes have an advantage to prevent overuse injuries.

## Damage to organs

An ultra-marathon can lead to further pathophysiological changes. Several studies revealed very different changes in laboratory values, with much of these changes being due to direct organ damage. Even a relatively short running distance can lead to considerable changes in biomarkers where the intensity or duration of the performance is proportional to the change in a specific marker (Bird et al., 2014). In other words, the body responds to the stress of an ultra-marathon with an “acute-phase reaction” (Fallon et al., 1999b; Fallon, 2001).

An ultra-marathon can lead to changes in biomarkers indicating a pathological process in specific organs or organ systems such as skeletal muscles, liver, kidney, etc. (Noakes and Carter, 1982; Nagel et al., 1990; Wu et al., 2004; Kim et al., 2007; Bürge et al., 2011; Shin et al., 2012; Bird et al., 2014; Jastrzebski et al., 2016); Table 9. These changes are usually temporary, depending on both the intensity and the duration of the performance and usually normalize after the race (Bird et al., 2014) mainly with a few days (Kłapcinska et al., 2013). When biomarkers change during an ultra-marathon, these changes are transient and there are no long-term or deleterious consequences (Wu et al., 2004; Bird et al., 2014). The changes affect both younger and older runners alike, regardless of their running experience (Jastrzebski et al., 2016). However, there are also biomarkers which remain unchanged despite high levels of physical stress (Fallon et al., 1999a).

**Table 9:** Changes in metabolites and hormones during an ultra-marathon.

**SKELETAL MUSCLE AND HEART MUSCLE**	
Creatin-Kinase	↑/↑↑
Creatin-Kinase-MB	↑
Myoglobin	↑
Cardiac troponins	↑
Lactate dehydrogenase	↑
**LIVER**	
Alkaline phosphatase	↑
Gamma glutamyltransferase	↑
Alanine aminotransferase	↑
Aspartat aminotransferase	↑
Bilirubin	↑
**KIDNEY AND FLUID METABOLISM**	
Creatinine	↑ / ↑↑
Sodium	↓/ = /↑
Potassium	↓
Calcium	↑ / =
Phosphate	↑
Protein	↓
Albumin	↓
**BLOOD AND BLOOD CELLS**	
Plasma volume	↑ / ↓
Erythrocytes	↓
Leucocytes	↑ / ↑↑
Haptoglobin	↑ / ↓
Iron	↑
Ferritin	↑
**INFLAMMATION**	
C-reactive protein	↑ / ↑↑
Erythrocyte sedimentation rate	↑
Interleukin-6	↑
Interleukin-8	↑
Interleukin-10	↑
Tumornekrosefaktor	↑
**HORMONE**	
Testosterone	↓
Cortisol	↑
Adrenocorticotrope hormone	↑
Noradrenaline	↑
Adrenaline	↑
Dopamine	↑
Growth hormone	↑
Prolactin	↑
Vasopression	↑
Copeptin	↑
Aldosterone	↑
Atrial natriuretic peptide	↑
N-terminales pro-Brain Natriuretic Peptide	↑
**FAT METABOLISM**	
Free fatty acids	↑
Cholesterol	↓
LDL-Cholesterol	↓


## Blood

An ultra-marathon can lead to hemolysis (Chiu et al., 2015) and it is assumed that hemolysis leads to a significant loss of erythrocytes (Robach et al., 2014). Hematocrit has been shown to decrease after an ultra-marathon (Cejka et al., 2012) and haptoglobin increases (De Paz et al., 1995; Chiu et al., 2015). Haptoglobin does not always increase after an ultra-marathon, possibly the length of the race is crucial. At least, after a 60-km ultra-marathon (Lippi et al., 2012b) and a 24-h ultra-marathon (Liu et al., 2018), there was a decrease in haptoglobin. In a 166-km mountain ultra-marathon, it has been shown that “stress anemia” only occurs by causing anemia due to expansion of plasma volume (Fallon et al., 1999a) and not due to a reduction in the volume of erythrocytes (Robach et al., 2014). The extent of hemolysis can be significantly reduced by appropriate training (Casoni et al., 1985) and a corresponding diet with antioxidants (Aaseth and Birketvedt, 2012).

The phenomenon of hemolysis in runners and ultra-marathoners has been reported (Lippi et al., 2012b; Fazal et al., 2017). Repetitive forceful foot striking can lead to blood cell lysis in the feet, resulting in a mild macrocytic anemia and intravascular haemolysis (Fazal et al., 2017). The decrease of haptoglobin after an ultra-marathon reflects a certain degree of haemolysis, but since the changes in red blood cells is not relevant, the foot-strike haemolysis is very modest or even clinically negligible (Lippi et al., 2012b). Most probably there is no cellular damage to the red blood cells. A study with marathon and ultra-marathoners showed that hemoglobin, erythrocytes and erythrocyte indices did not change during the race (Banfi et al., 2004). In a 24-h ultra-marathon, red blood cells, hemoglobin, hematocrit, and mean cell hemoglobin decreased (Liu et al., 2018). Ultimately, the suspected anemia of the ultra-runner is just a “dilute anemia” (Dickson et al., 1982). The reticulocytes increase after a 6-day ultra-marathon (Fallon and Bishop, 2002), possibly as an indication that a longer ultra-marathon leads to some damage to the erythrocytes. As part of iron metabolism, ferritin increases (Liu et al., 2018) and transferrin saturation decreases (Kasprowicz et al., 2013). In the case of ferritin it could be shown that the value is increased due to the training and can remain elevated after an ultra-marathon up to 2 weeks (Dickson et al., 1982). Although hemolysis with a cellular damage of the red blood cells seems possible, it will not be of relevance. A decrease in hemoglobin is only due to plasma volume expansion.

## Hormones

During an ultra-marathon, some characteristic changes in specific hormones are evident (Pestell et al., 1989; Fournier et al., 1997), with the hypothalamic pituitary axis usually changing (Wittert et al., 1996; Table 9). An ultra-marathon leads to an increase in cortisol (Fournier et al., 1997), catecholamines (Pestell et al., 1993), and growth hormone (McKechnie et al., 1982) as well as a drop in testosterone (Fournier et al., 1997) where the decrease in testosterone is related with a decrease in libido (Longman et al., 2018).

The length of an ultra-marathon seems to have an influence on the change in hormones. In a run from Sydney to Melbourne over 1,000 km, an increase of norepinephrine, epinephrine, dopamine and adrenocorticotropic hormone was found. No changes were found for ß-endorphin, growth hormone, prolactin, testosterone, cortisol and cortisol-binding globulin. After the ultra-marathon, catecholamines increased and growth hormone, prolactin and cortisol increased (Pestell et al., 1989). Obvioulsy, the ultra-marathon leads to a chronic physical stress leading to an adjustment of stress hormones with a continuous increase (Pestell et al., 1989). The intensity is also crucial. Higher cortisol levels were found after an ultra-marathon when the athlete was competing at a high running speed (Tauler et al., 2014).

Since not only men but also more and more women run ultra-marathons, the changes of the female hormones were also investigated. There was a marked increase in estradiol after an ultra-marathon (Copeland and Verzosa, 2014). There is also a change in neurotransmitters, such as an increase in serotonin, a drop in tryptophan and an increase in ß-endorphins (Agawa et al., 2008; Table 9). In summary, male ultra-marathoners must be aware that increased levels of cortisol and suppressed levels of testosterone might become counterproductive.

## Skeletal muscle damage

The term “skeletal muscle damage” applied to ultramarathon refers to the race-induced muscle pain that results not from fatigue but from muscle injury (Damas et al., 2018). An ultra-marathon has a considerable influence on the skeletal muscles (Kim et al., 2007), with the down passages causing the greatest muscle damage during the run (Koller et al., 1998) where a run of 330 km with an elevation gain of 24,000 m leads to a measurable inflammatory reaction and swelling of the thigh musculature (Andonian et al., 2016). Accordingly, skeletal muscle damage consists in a major concern for ultramarathon runners.

Studies in ultramarathon have used several blood markers to evaluate skeletal muscle damage. Skeletal muscle damage is well visible on specific myocellular metabolites increasing in blood, such as myoglobin (Bird et al., 2014; Jastrzebski et al., 2016), lactate dehydrogenase (Noakes and Carter, 1982; Kanter et al., 1986; Chiu et al., 2013; Bird et al., 2014; Shin et al., 2014; Jastrzębski et al., 2015a,b), and creatine kinase (Kanter et al., 1986; Suzuki, 2002; Chiu et al., 2013; Jee et al., 2013; Bird et al., 2014; Carmona et al., 2015; Jastrzębski et al., 2015b).

**Figure 4:**
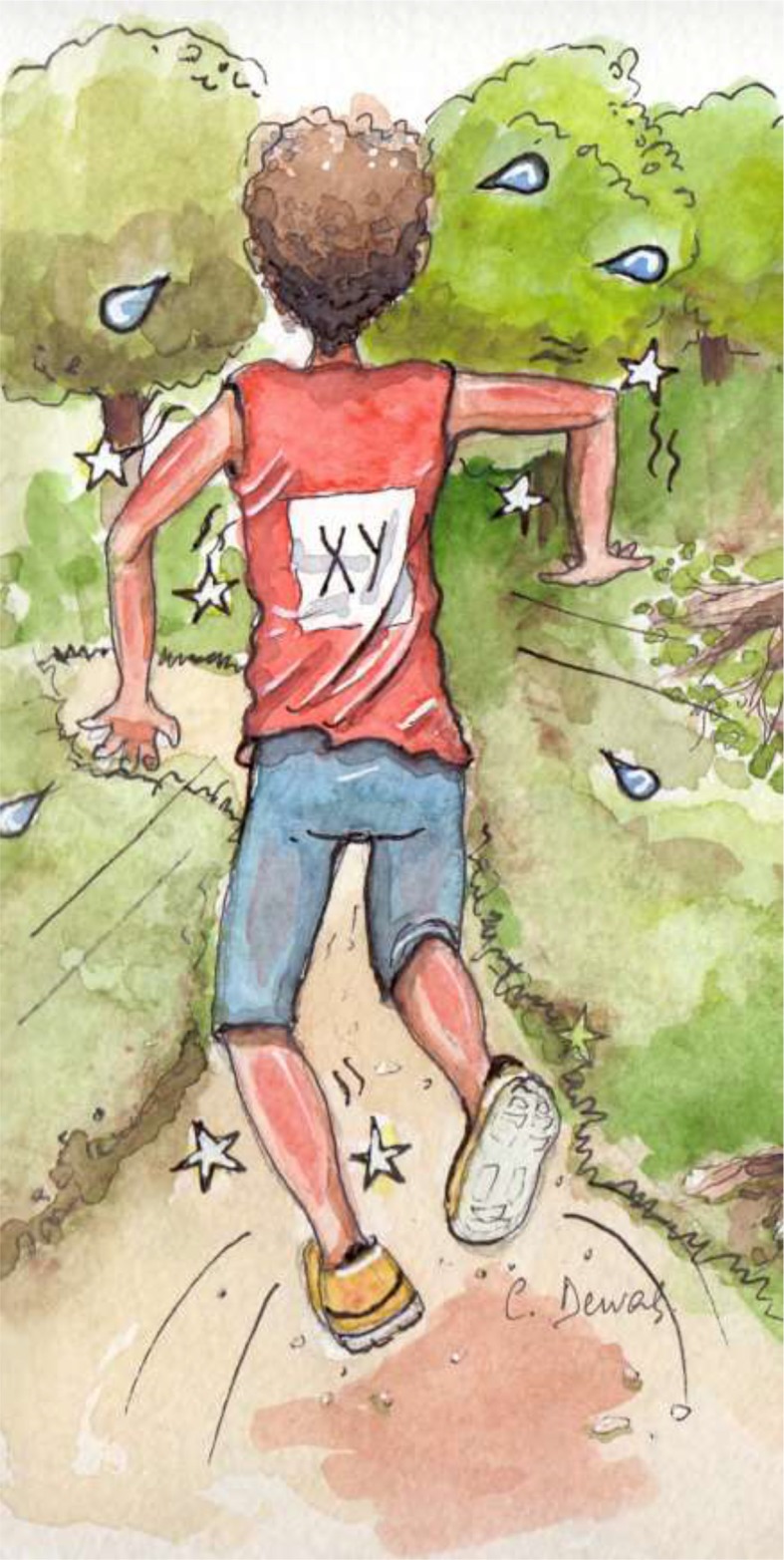
Muscle pain can accompany muscle damage occurring during an ultra-marathon. Figure by Céline Dewas.

The creatine kinase as a muscular enzyme is very suitable to document muscular damage due to an ultra-marathon. The eccentric load, such as in a mountain ultra-marathon, can lead to a significant increase in creatine kinase (Noakes et al., 1983; Frey et al., 1994) and pronounced muscle soreness (Figure 4). The highest activity of creatine kinase is measured about 1 h after the completion of an ultra-marathon (Carmona et al., 2015) but may still be highest at ~36–72 h after the race (Bird et al., 2014). The increase in creatine kinase after an ultra-marathon can sometimes be grotesque (Noakes and Carter, 1982; Kim et al., 2007, 2009) and seems to increase with increasing race distance (Table 10). An ultra-marathon over 308 km leads to a significantly higher creatine kinase than an ultra-marathon over 100 km (Yoon et al., 2016). In a 200-km ultra-marathon, creatine kinase increased to 35 times the baseline and remained elevated until 5 days after the race (Kim et al., 2009). In another 200-km ultra-marathon, the creatine kinase even rose to 90 times the initial value (Kim et al., 2007). In the “Badwater,” creatine kinase can increase up to ~27,951 U/l (Roth et al., 2007). And in the “Western States Endurance Run,” 216 (66%) of 328 finishers had creatine kinase concentrations of 1,500 U/l to 264,300 U/l and 13 (6%) of the finishers had values of more than 100,000 U/l (Hoffman et al., 2012a).

**Table 10:** Pre- and post-race CK-values from different races, sorted by the length of the races.

**Race**	**Length/Duration**	**Change in CK**	**References**
Swiss Alpine Marathon	67 km, altitude difference of 2,300 m	From 600 U/l to 28,000 U/l	Frey et al., 1994
100 km Lauf Biel	100 km	From 157.8 ± 74.5 U/l to 3,861.5 ± 2,357.8 U/l	Knechtle et al., 2011a
100 km Lauf Biel	100 km	From 168.3 ± 61.7 U/l to 4,582 ± 3,150 U/l	Knechtle et al., 2012a
Not specified	100 km	From 127.20 ± 31.12 U/l to 388.40 ± 208.05 U/l	Yoon et al., 2016
Not specified	100 km	From 145.87 ± 99.1 U/l to 1,532.75 ± 981.91 U/l	Jastrzębski et al., 2015b
Flexpower Cup National 100-Km Ultra-Marathon in Taiwan	100 km	From 172.3 ± 91.7 U/l to 4,274.8 ± 5,903.9 U/l	Chiu et al., 2013
Western States Endurance Run 2012	100 miles	From 117 U/l (23–140) to 17,965 U/l (10,975–24,956)	Kupchak et al., 2013
Not specified	166-km mountain ultra-marathon (MUM) with 9500 m of positive and negative elevation change	From 144 ± 94 U/l to 13,633 ± 12,626 U/l	Millet, 2011
Not specified	180 km	To 20.000 U/l	Aaseth and Birketvedt, 2012
Not specified, case study	227 km in a 24-h ultra-marathon	447 U/l to 60,060 U/l	Niemela et al., 1984
Tor des Geants	330-km trail run with 24000 m of positive and negative elevation change	To 3,719 ± 3,045 U/l	Saugy et al., 2013
Run from West coast to East coast of Korea	308 km	From 153.9 ± 68.8 U/l to 5,281.5 ± 2,388.4 U/l	Shin et al., 2014
Continuous race from Kanghwado to Kangneung, South Korea	308 km	To 5,100.37 ± 2,121.33 U/l	Shin and Lee, 2013
Not specified	308 km	From 153.53 ± 69.00 U/l to 5,270.06 ± 2,575.78 U/l	Yoon et al., 2016
Not specified	308 km with measurements after 100, 200, and 308 km	To 1,127.2 ± 507.9 U/l, 5133.8 ± 2,492.7 U/l and 4,958.4 ± 2,087.9 U/l at 100, 200, and 308 km, respectively	Kim et al., 2014
Not specified	24-h ultra-marathon	From 249.2 ± 136.1 U/l to 17,502 ± 15,040 U/l	Waśkiewicz et al., 2012

The increase in creatine kinase seems to be dependent on the fitness level of the runner (Noakes and Carter, 1982). Top ultra-marathoners have a lower creatine kinase before the start than slower runners, and after the run have significantly a lower creatine kinase than slower finishers (Suzuki, 2002). Ultra-marathoners with a creatine kinase higher than 500 mU/ml before the start felt tired and rather dropped out of the race (Suzuki, 2002). Important for athletes and coaches is the fact that the majority of athletes with significantly elevated creatine kinase levels are asymptomatic and do no require major medical attention (Magrini et al., 2017).

The extent of the skeletal muscle damage is clearly reflected in a severely limited muscular function after an ultra-marathon (Davies and Thompson, 1986) where standardized jumping exercises are restricted for ~18 days after an ultra-marathon (Chambers et al., 1998). The extensive muscle damage seems to lead to a decrease in muscle mass. Several studies have shown that an ultra-marathon leads to a significant reduction in skeletal muscle mass (Knechtle et al., 2011a, 2012a,e). Considering the relationship between skeletal muscle mass and muscle strength, it might be assumed that the post-race decrease in muscle strength would be partially attributed to the decrease of skeletal muscle mass.

Potential therapeutic options were investigated to reduce skeletal muscle damage during ultra-marathon running or to improve recovery after the race. An option was to reduce the decrease in skeletal muscle damage or to reduce the increase in metabolites of skeletal muscle damage by consumption amino acids during an ultra-marathon (Knechtle et al., 2012a). However, BCAA-supplementation before and during a 100-km ultra-marathon did not affect race performance and biomarkers of skeletal muscle damage (Knechtle et al., 2012a) and muscle pain (Knechtle et al., 2011a). It has also been investigated whether the use of a non-steroidal anti-inflammatory drug (NSAID) leads to reduced skeletal muscle damage. However, the use of diclofenac in a mountain ultra-marathon showed no effect on the increase in creatine kinase (Frey et al., 1994). The 100-mile “Western States Endurance Run” also showed that the intake of ibuprofen had no effect on skeletal muscle damage and pain sensation. Intake of ibuprofen leads to an increase in inflammatory markers such as C-reactive protein and interleukins (Nieman et al., 2006). The reason to consume NSAIDs in ultra-marathon running is explained by osteo-articular pain or to prevent pain. Ultra-marathoners consuming NSAIDS are predominantly motivated by their personal achievement (Didier et al., 2017). The intake of antioxidants does not seem to have any influence on skeletal muscle damage (Mastaloudis et al., 2004a, 2006). A diet with antioxidants leads to a significant reduction in skeletal muscle damage and increase in creatine kinase (Aaseth and Birketvedt, 2012).

The best option for a fast recovery after an ultra-marathon is appropriate training. In finishers in a 100-mile ultra-marathon, runners with lower post-race creatine kinase levels were better prepared for the race (Hoffman et al., 2017a). The extent of skeletal muscle damage can be significantly reduced by appropriate training with high training volumes (Hoffman et al., 2016) and long race experience (Noakes and Carter, 1982). It is well-known that the training for an ultra-marathon leads to characteristic changes and adaptations in the muscle fiber. As an adaptation to training, the mitochondria are very close to the capillaries, there is a proliferation of capillaries as well as an increase and enlargement of type I fibers (Crenshaw et al., 1991). Manual therapy and intermittent pneumatic compression are recovery methods used by endurance athletes with little evidence supporting effectiveness. In a controlled study with ultra-marathoners, both treatments reduced muscular fatigue scores acutely after treatment following the race and on post-race day 1. In addition, manual therapy improved muscle pain and soreness acutely following the race (Heapy et al., 2018). In summary, each ultra-marathon leads to skeletal muscle damage. Although levels of creatine-kinase can rise up to more than 100,000 U/l, this damage is reversible. The best prevention is appropriate training in the pre-race preparation.

## Bone

Compared to other physiological systems and organs of human body, acute and chronic race-induced adaptations of bone in ultramarathon runners have been less studied. In addition to skeletal muscle, also bone appears to be damaged during an ultra-marathon (Kerschan-Schindl et al., 2009; Sansoni et al., 2017). During the “Spartathlon,” there is a direct influence on metabolites of bone metabolism leading to an increased bone resorption and a reduced bone formation (Kerschan-Schindl et al., 2009). In a mountain ultra-marathon, a correlation between the high energy consumption and bone damage with a decrease in osteocalcin was demonstrated (Sansoni et al., 2017). This could lead to a decrease in bone density leading to osteopenia and to osteoporosis after prolonged exposure. Furthermore, bone mineral density of women ultramarathoners has been related to menstrual function, where low lumbar spine bone mineral density has been observed in runners with history of oligo/amenorrhea (Micklesfield et al., 1995). Accordingly, it has been suggested that postmenopausal ultra-marathon runners were at high risk for large losses in bone mass (Folscher et al., 2015). Consequently, special attention is needed on the bone health of women ultramarathon runners.

## Heart

As ultramarathon is a prolonged endurance exercise relying on aerobic energy transfer system, it is not surprising that a large body of the literature has focused on the exercise physiology of cardiovascular system. Due to the increase in creatine kinase, several studies investigated a potential heart damage through an ultra-marathon due to an increase in cardiac biomarkers such as creatine kinase, creatine kinase-MB, cardiac troponin I and N-terminal pro-brain natriuretic peptides after an ultra-marathon (Table 9; Roth et al., 2007; Kim et al., 2014; Christensen et al., 2017). A 90-km ultra-marathon also leads to a an increase in C-reactive protein as has been reported for heart attack patients (Strachan et al., 1984). However, the changes in these biomarkers are all transient (Christensen et al., 2017).

Running speed and the length of an ultra-marathon are important for potential changes of markers of cardiac damage. An ultra-marathon over 100 km leads to a significantly higher creatine kinase than an ultra-marathon over 308 km (Yoon et al., 2016). It is well-known that high-intensity endurance exercise leads to biochemical changes that may indicate heart damage (Salvagno et al., 2014). The highly sensitive cardiac troponin I increases after an ultra-marathon (Salvagno et al., 2014) where faster runners show a higher increase in cardiac troponin I than slower runners (Musha et al., 1997; Khodaee et al., 2015). The increase in cardiac troponin I can partially be dramatic. In the “Supermaratona dell'Etna” covering 43 km from sea level to 2850 m, mean cardiac troponin I increased by +900% (Da Ponte et al., 2018).

In men in a 308-km ultra-marathon, a normal creatine kinase-MB mass index (< 5.0 ng/ml) and no increase in cardiac troponin I could be detected and it was assumed that there had been no myocardial damage despite an increase in the creatine kinase-MB (Kim et al., 2014). Even in runners competing in “Badwater,” no structural damage to the myocardium could be demonstrated (Roth et al., 2007). In a 50- and 100-mile ultra-marathon, an increased activity of serum creatine kinase-MB could be detected in ~80% of the runners, with no evidence of myocardial damage in myocardial scintigraphy (Matin et al., 1983). Apart from creatine kinase, also specific cardiac hormones increase during an ultra-marathon. The hormones N-terminal brain natriuretic peptide (BNP) (Ohba et al., 2001; Hew-Butler et al., 2008a,b; Tchou et al., 2009) and atrial natriuretic peptide (ANP) (Ohba et al., 2001) were increased post-race.

It is very controversial whether an ultra-marathon leads to a damage of the heart. A study of 100-mile ultra-marathoners showed that the race induced changes in the heart. In the electrocardiogram, changes occurred in the right heart (Lord et al., 2015). The right heart leads showed a change in the electrocardiogram after the run compared to the electrocardiogram before the run for the P-wave, the ST-segment and the T-wave (Lord et al., 2016a).

A structural change of the heart muscle by an ultra-marathon could be detected by the use of echocardiography. Echocardiography can detect a reduction of the left (Niemela et al., 1984; Krzeminski et al., 2016; Maufrais et al., 2016) as well as the right ventricular (Oxborough et al., 2011; Lord et al., 2015, 2016b; Maufrais et al., 2016; Rothwell et al., 2018) function after an ultra-marathon. The changes in cardiac troponin I during an ultra-marathon are inversely associated with left ventriculare ejection fraction determined with echokardiography (Christensen et al., 2017).

The length of the ultra-marathon is important whether a change in the left and/or right ventricular function occurs. After the 2-day “Lowe Alpine Mountain Marathon,” both a systolic and diastolic dysfunction of the left ventricle could be detected. Humoral markers of myocardial damage were increased and the increase in cardiac troponin was considered to be associated with a minimal myocardial damage (Shave et al., 2002). After a 24-h ultra-marathon, two out of 20 runners showed a slight increase in cardiac troponins and echocardiography showed a decrease in left ventricular ejection fraction in one of the two runners (Passaglia et al., 2013). After an 89-km ultra-marathon, there was a reduced function of the left and right ventricles (Chan-Dewar et al., 2010). In a 4-h run, there was a decrease in the activity of the MIBG (131-J meta-iodo-benzylguanidine) in the myocardium, and the extent of activity decrease correlated with the distance covered during the run (Estorch et al., 1997). In a 160-km ultra-marathon, no correlation between the decrease in left ventricular function and the change in cardiac biomarkers could be demonstrated (Scott et al., 2009). During a 24-h ultra-marathon, the left ventricular function decreased during the last 6 h of the race where the function normalized within a few days after the race (Niemela et al., 1984). Another study showed that echocardiographic changes returned to normal within 1 day after the run (Dávila-Román et al., 1997). In some instances, an ultra-marathon leads to no echocardiographic changes. For runners in the “Western States Endurance Run,” echocardiographic findings were normal (George et al., 2011). Therefore, it is at the moment difficult to conclude that an ultra-marathon leads to substantial heart damage.

Although an ultra-marathon leads to a limitation of the left ventricular function and an increase in certain cardiac biomarkers, the mechanism behind these changes is unknown (Scott et al., 2009). An important aspect is also that the endurance training leads to an adjustment of the left ventricle in the sense of a left ventricular hypertrophy (Nagashima et al., 2003; Szauder et al., 2015). The size of the left ventricle is an important predictive variable for the performance of an ultramarathon (Nagashima et al., 2006). As women progressively compete in ultra-marathon, it has also been investigated whether a sex differences exist. In a 100-km and a 100-mile ultra-marathon, both men and women were examined and no difference was found in the echocardiographic changes between the sexes (Cote et al., 2015).

It is believed that elevated cholesterol is a cardiovascular risk factor with an increased morbidity and mortality. An ultra-marathon could be beneficial for reducing elevated cholesterol (Thompson et al., 1982; Kaminsky et al., 1988; Wu et al., 2004; Sapounakis et al., 2010). There has been shown a reduction in triglycerides and total cholesterol after a 24-h ultra-marathon, but no change in low density lipoprotein cholesterol and high density lipoprotein cholesterol (Emed et al., 2016). In another study, a reduction in total cholesterol, low density lipoprotein cholesterol and triglycerides was demonstrated after an ultra-marathon (Kaminsky et al., 1989). Only training lowers low density lipoprotein cholesterol in an ultra-marathon (Tomaszewski et al., 2004). Runners preparing for an ultra-marathon experience a decrease in total and low density lipoprotein cholesterol (Sapounakis et al., 2010). Obviously, training for and competing in an ultra-marathon might be considered as preventive for cardiovascular diseases.

## Digestive tract

**Figure 5:**
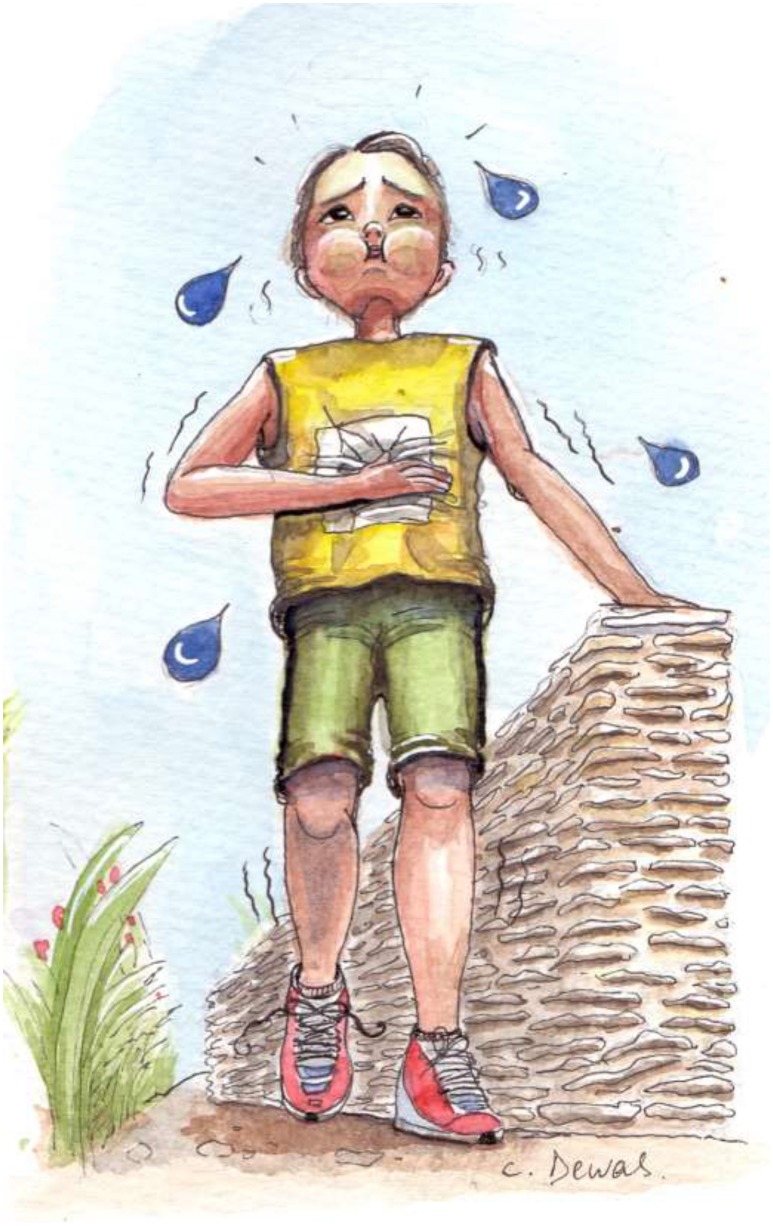
Nausea is the most frequently mentioned digestive problem in ultra-marathon runners. Figure by Céline Dewas.

The physiology and pathophysiology of digestive tract in ultramarathon runners attracts widespread interest due to the abovementioned large energetic demands of training and competititon. Digestive tract plays a critical role in delivering nutrients during endurance exercise; thus, the occurrence of digestive problems might limit performance (Jeukendrup, 2017). Ultra-marathoners often suffer from digestive problems (Rehrer et al., 1992) and gastrointestinal bleeding after an ultra-marathon is not uncommon (Baska et al., 1990) where often occult bleeding occurs (Chiu et al., 2015). Symptoms of the lower gastrointestinal tract correlate with gastrointestinal bleeding (Baska et al., 1990). In a 100-mile ultra-marathon, ~35% (Stuempfle and Hoffman, 2015) and in a mountain ultra-marathon ~43% of runners complained about digestive problems (Rehrer et al., 1992). In some cases as many as 80% and more of the finishers complain of digestive problems (Wardenaar et al., 2015; Stuempfle et al., 2016), with nausea being mentioned most frequently (Figure 5; Stuempfle et al., 2016). Digestive problems are one of the main reasons why ultra-runners have to give up an ultra-marathon (Hoffman and Fogard, 2011). Up to 90% of runners who give up an ultra-marathon complain of nausea (Stuempfle and Hoffman, 2015).

A possible reason for these digestive problems could be that an ultra-marathon changes the motility of the esophagus (Simons and Kennedy, 2004). Endotoxemia seems to be a major cause of digestive problems, while factors such as hyperthermia, dehydration and nutrition are barely causative (Stuempfle et al., 2016). Experience seems to be important. Runners with digestive problems during an ultra-marathon have less training km and shorter training runs (Glace et al., 2002). From a pathomechanistic point of view, endotoxins and pro-inflammatory cytokines increased in a 24-h ultra-marathon and this increase led to a counter-regulatory anti-inflammatory reaction (Gill et al., 2015a,b).

From a therapeutic point of view, it seems very difficult to treat gastrointestinal problems. The intake of sodium during a 100-mile ultra-marathon definitely has no influence on nausea and vomiting (Hoffman and Stuempfle, 2016a). The best way to prevent digestive problems is to eat what you like most during an ultra-marathon (Moran et al., 2011). The high-fat foods and increased fat intake during an ultra-marathon seem to cause significantly less digestive problems (Stuempfle et al., 2013) and ingesting a proton pump inhibitor before an ultra-marathon significantly reduces the risk of gastrointestinal bleeding during the run (Thalmann et al., 2006). Regarding digestive problems in ultra-marathon running, the prevalence is very high and the only option is prevention by eating what you like most during an ultra-marathon.

## Liver

Regular physical activity has a positive effect on liver function. On the other hand, a very long endurance exercise—especially under difficult climatic conditions (Carvalho et al., 2016)—can become a problem for the liver (Shephard and Johnson, 2015). Especially longer runs at low intensity seem to be more of a problem for the liver (Shin et al., 2016). There may be a detectable impairment in liver function during an ultra-marathon (De Paz et al., 1995; Chiu et al., 2013; Bird et al., 2014; Shin et al., 2014; Table 9).

An ultra-marathon can lead to an increase in gamma-glutamyltransferase (De Paz et al., 1995; Shin et al., 2014), alanine aminotransferase (De Paz et al., 1995; Chiu et al., 2013; Bird et al., 2014; Kupchak et al., 2014; Jastrzębski et al., 2015b), aspartate aminotransferase (De Paz et al., 1995; Chiu et al., 2013; Bird et al., 2014; Kupchak et al., 2014; Jastrzebski et al., 2015; Jastrzębski et al., 2015b), alkaline phosphatase (Noakes and Carter, 1982; Wu et al., 2004; Kupchak et al., 2014), and bilirubin (De Paz et al., 1995; Fallon et al., 1999a; Wu et al., 2004; Bird et al., 2014; Shin et al., 2014; Chou et al., 2016; Jastrzebski et al., 2016). These changes are dependent on the intensity and/or duration of the physical performance and generally normalize within a few days after the race (Wu et al., 2004; Bird et al., 2014).

However, it can happen in very rare cases that an ultra-marathon can lead to pronounced liver damage (Heneghan et al., 2014; Carvalho et al., 2016). In a young runner in a 62-km ultra-marathon a case was described with a heat stroke. As a result, pronounced rhabdomyolysis and hypoxic hepatitis with multiple organ failure, including fulminant liver failure, resulted in intensive care measures. Later, an emergency hepatectomy and orthotopic liver transplantation had to be performed (Heneghan et al., 2014). In another case, a 25-year-old man had hyperthermia with neurological restrictions during an ultra-marathon. In the further course, acute liver failure occurred and the patient had to be monitored intensively but recovered again (Carvalho et al., 2016). Regarding a potential liver damage, the increase in biomarkers of liver damage is reversible and a serious liver damage is very seldom to expect.

## Kidney

During ultra-marathon running, a damage to the kidney with an impaired renal function is quite often observed (Boulter et al., 2011). The prevalence of an acute kidney injury in ultra-marathon running is nearly 50% of all runners (Lipman et al., 2017).

From a pathophysiological point of view, the skeletal muscle damage leads to an influx of muscle proteins, such as myoglobin, into the bloodstream. In certain circumstances, such as pronounced dehydration or heat, there may be a marked accumulation in the kidney with consequent kidney damage (Schiff et al., 1978; Uberoi et al., 1991; Clarkson, 2007).

Regarding fluid and electrolyte metabolism, an ultra-marathon leads to quite characteristic changes. It seems that short and fast ultra-marathons are more likely to cause a kidney problem than longer ultra-marathons at lower speeds (Shin et al., 2016). An ultra-marathon leads to an increase in creatinine (Schiff et al., 1978; Uberoi et al., 1991; Clarkson, 2007; Lipman et al., 2017), urea (Shin et al., 2016), uric acid (Schiff et al., 1978; Uberoi et al., 1991), sodium (McKechnie et al., 1982; Bürge et al., 2011; Cejka et al., 2012), potassium (Uberoi et al., 1991; Kłapcinska et al., 2013; Jastrzebski et al., 2016), calcium (Fallon et al., 1999a; Kłapcinska et al., 2013) and phosphate (Fallon et al., 1999a). Furthermore, there is also a characteristic change in hormones of water and electrolyte metabolism such as vasopressin (Hew-Butler et al., 2008a, 2011; Rogers et al., 2011), copeptin (Hew-Butler et al., 2011; Lippi et al., 2015), oxytocin (Hew-Butler et al., 2008a) and aldosterone (Hew-Butler et al., 2011; Table 9).

An ultra-marathon often leads to a temporary reduction in renal function (Lippi et al., 2012a; Lipman et al., 2014, 2016; Hou et al., 2015; Mrakic-Sposta et al., 2015; Hoffman and Weiss, 2016), although in some cases all runners tested suffer from renal impairment (Kao et al., 2015). In rare cases, there is evidence of a considerably impaired renal function (Irving et al., 1990). Generally, the kidney function recovers within one (Kao et al., 2015) to a few days (Kallmeyer and Miller, 1993). In a multi-stage ultra-marathon, the kidney function often recovers until the next morning's start (Lipman et al., 2014). It can also happen that a dialysis-dependent renal insufficiency develops during an ultra-marathon (Uberoi et al., 1991). Even if a runner experiences a restriction of renal function during an ultra-marathon, another ultra-marathon does not necessarily lead to an additional restriction of kidney function (Irving et al., 1990; Hoffman and Weiss, 2016). It has been shown that certain people rather suffer a kidney problem and/or a certain behavior can preferably lead to a kidney problem.

Risk factors for kidney damage during an ultra-marathon are female sex, a low body weight and a significant body weight loss during the run (Lipman et al., 2016). Other risk factors include severe muscle damage with rhabdomyolysis, dehydration, hypotension, hyperuricemia, hyponatraemia, poor competition, and the use of NSAIDs (Seedat et al., 1989; Clarkson, 2007; Boulter et al., 2011). In ultra-marathoners, increased rates of acute kidney injury were found in those who took ibuprofen (Lipman et al., 2017).

From a diagnostic point of view, the test strip method under field conditions could serve well to detect kidney damage due to an ultra-marathon. In a certain percentage of finishers, microhematuria is a sign of kidney damage (Kallmeyer and Miller, 1993). In 100-mile ultra-marathoners, blood and urine levels were measured and compared upon arrival at the finish line. In ultra-marathoners with kidney damage detected by creatinine levels in the blood, the test strip showed very high specificity and sensitivity, protein, blood and increased urine specific gravity (Hoffman et al., 2013b). Damage to the kidneys occurs very often in ultra-marathon running; however, the kidney function recovers well within a few days. Similarly to the liver, serious kidney damage is very seldom to expect.

## Immune system

It is well-known that intense physical stress leads to tissue damage (McKune et al., 2005). Experienced ultra-marathoners experience also a change in the concentration of immunoglobulins after an ultra-marathon, which might have an impact on the health of finishers (McKune et al., 2005). An ultra-marathon seems to lead to an acute inflammatory reaction with classical laboratory chemical changes (Kasprowicz et al., 2013; Shin and Lee, 2013). The stress-related inflammation affects the bone marrow and leads to an increased leukocyte turnover (Spiropoulos et al., 2010). Markers of an inflammatory process include leucocytes (Jee et al., 2013; Bird et al., 2014; Chiu et al., 2015; Jastrzebski et al., 2016; Zakovska et al., 2017), C-reactive protein (Kim et al., 2009; Waśkiewicz et al., 2012; Kasprowicz et al., 2013), ferritin (Chiu et al., 2015), TNF-α (Nieman et al., 2000; Simons and Kennedy, 2004; Jee and Jin, 2012; Chiu et al., 2013, 2015), blood sedimentation reaction, iron (Fallon, 2001), y-interferon (Simons and Kennedy, 2004), Interleukin 1 receptor antagonist (Nieman et al., 2000), Interleukin-1b (Simons and Kennedy, 2004; Gill et al., 2015b), Interleukin-8 (Nieman et al., 2000; Gill et al., 2015b), Interleukin-10 (Nieman et al., 2000; Simons and Kennedy, 2004; Gill et al., 2015b) and Interleukin-6 (Fallon et al., 1999a; Waśkiewicz et al., 2012; Kasprowicz et al., 2013).

There seems to be an association between the stress on the immune system and skeletal muscle damage. In 100-km ultra-marathoners, an association between the markers of the acute inflammation of the organism (i.e., neutrophils, immature neutrophils, platelets, and monocytes) and the markers of muscle damage (i.e., CK, platelets, and LDH) could be demonstrated (Zakovska et al., 2017).

Ultra-marathoners often suffer from upper respiratory infections after a race (Peters and Bateman, 1983; Peters et al., 1993). It has been demonstrated that ultra-marathoners are more likely to suffer from an infection than runners over shorter distances (Castell, 1996). The infections can already occur 4 weeks before the start and 7–14 days after the end of the race (Peters et al., 2010). Regarding the frequency of infections, ~25–30% of runners are affected by an ultra-marathon (Peters and Bateman, 1983; Nieman et al., 2003). In the “Two Oceans Marathon” held in Cape Town, South Africa, more than 30% of runners suffered from upper respiratory tract infections, mainly affecting the faster runners (Peters and Bateman, 1983). Often, faster ultra-marathoners have more infections than slower ultra-marathoners (Peters et al., 2004) while slower ultra-marathoners have the frequency of infections in the range of persons in a control group (Peters and Bateman, 1983). It has been shown that a 24-h ultra-marathon leads to a decrease in salivary flow, a decrease in salivary IgA, and a decrease in the secretion of salivary lysozyme (Gill et al., 2013, 2014).

Both the frequency and the severity of an infection after an ultra-marathon can be favorably influenced. The concentration of glutamine in the blood is reduced by up to ~20% after an ultra-marathon (Castell and Newsholme, 1997). Intake of glutamine before and after an ultra-marathon reduces the frequency of infections (Castell, 1996; Castell and Newsholme, 1997, 1998). In addition to glutamine, vitamin C has beneficial effects on upper airway infections in ultra-marathoners (Peters, 1997). The regular intake of vitamin C can improve the resistance to the increased occurrence of upper respiratory tract infections and—if an infection nevertheless occurs—somewhat reduce the severity of the infection (Peters et al., 1993). Even if vitamin C has an influence on an infection, the intake of vitamin C does not lead to a change in various infection parameters such as immune cells, interleukins, or interferon (Nieman et al., 2002). The amount of vitamin C intake seems important. In runners who successfully completed the “Comrades Marathon,” the daily intake of 1,500 mg of vitamin C in the week before the competition resulted in a significantly lower increase in interleukins than the daily intake of 500 mg (Nieman et al., 2000). A higher amount of vitamin C also leads to low increases in cortisol and adrenaline during the race (Peters et al., 2001a,b).

In very rare cases, an infection after an ultra-marathon can lead to quite disastrous dimensions. In a 51-year-old runner, after a multi-stage ultra-marathon, a necrotizing soft tissue infection of both feet with septicemia occurred. A transmetatarsal amputation had to be performed on the left and a femoral amputation on the right side (Huang et al., 2014).

An ultra-marathon seems to have a positive influence on the immune system (Schobersberger et al., 2000). There is a correlation between regular endurance training and an increase in natural killer cells, a subset of lymphocytes and this increase leads to a favorable influence on the natural immune system (Francavilla et al., 2005). Even if it is clear that an ultra-marathon reduces the concentration of immunoglobulins, endurance exercise with moderate intensity will increase the concentration of immunoglobulins and reduce the risk of infection (Nieman and Nehlsen-Cannarella, 1991).

An ultra-marathon seems to lead to a depression of the immune function with an increased prevalence of infections of the upper respiratory tract. Most probably the intensity of running is crucial since running at moderate intensity seems to increase the concentration of immunoglobulins with a reduced risk of infections.

## Are ultra-marathons beneficial for health?

Considering the increased exercise-induced stress for most physiological systems of human body as demonstrated in the previous sections of this review, one might assume that the completion of ultra-marathons must be very health-damaging. Undoubtedly, the completion of an ultra-marathon has no immediate health benefits. However, with regards to the length of the telomeres, training for ultra-marathons seems to have a certain positive effect in endurance athletes (Borghini et al., 2015). Particularly, regular endurance training seems to have a protective effect on telomere length and should slow down the aging process (Denham et al., 2013). On the other hand, an extreme ultra-marathon such as “Tor des Géants” (330 km, 24,000 m vertically) leads to shortening of telomeres, probably due to oxidative damage to the DNA (Borghini et al., 2015). However, it does not necessarily take such a tremendous run as the “Tor des Géants”; even a 50-km run might provoke DNA damage (Mastaloudis et al., 2004b). The production of serum reactive oxygen species increases during an ultra-marathon indicating that the antioxidant system restricts the production of serum reactive oxygen species (Hattori et al., 2009). Thus, it might be supported that the overall effect of ultra-marathon on health is positive due to the beneficial role of endurance exercise.

## Conclusions

In summary, considering the increased number of participants in ultra-marathons, the findings of the present review would have practical applications for a large number of sports scientists and sports medicine practitioners working in this field. Ultra-marathoners seem to be aware of the potential harmful influence on health, but also know that the side effects of ultra-marathon running are reversible within a few days. It should be highlighted that only one review had been conducted previously concerning only ultra-marathoners which focused on a very specific topic (neuromuscular fatigue) (Millet, 2011). Other reviews examined musculoskeletal injuries of both ultra-marathoners and other distance runners (e.g., marathon and shorter distances) (Kluitenberg et al., 2015; Videbæk et al., 2015). Thus, the present review is the most comprehensive review ever conducted on physiology and pathophysiology in ultra-marathon.

## Author contributions

BK and PN collected the studies for the review and drafted the manuscript.

### Conflict of interest statement

The authors declare that the research was conducted in the absence of any commercial or financial relationships that could be construed as a potential conflict of interest.
